# The archaeal and bacterial community structure in composted cow manures is defined by the original populations: a shotgun metagenomic approach

**DOI:** 10.3389/fmicb.2024.1425548

**Published:** 2024-11-01

**Authors:** Vanessa Romero-Yahuitl, Karla Estephanía Zarco-González, Ana Lilia Toriz-Nava, Mauricio Hernández, Jesús Bernardino Velázquez-Fernández, Yendi E. Navarro-Noya, Marco Luna-Guido, Luc Dendooven

**Affiliations:** ^1^Laboratory of Soil Ecology, Department of Biotechnology and Bioengineering, Cinvestav, Mexico City, Mexico; ^2^Laboratorio de Interacciones Bióticas, Centro de Investigación en Ciencias Biológicas, Universidad Autónoma de Tlaxcala, Tlaxcala, Mexico; ^3^Departamento de Biología Celular y Genética, Escuela de Biología, and Instituto de Investigaciones en Microbiología, Facultad de Ciencias, Universidad Nacional Autónoma de Honduras, Tegucigalpa, Honduras

**Keywords:** archaeal community structure, bacterial community structure, methanogens, methanotrophs, methylotrophs, archaeal and bacterial nitrifiers, genes involved in the N cycle

## Abstract

**Introduction:**

Organic wastes are composted to increase their plant nutritional value, but little is known about how this might alter the bacterial and archaeal community structure and their genes.

**Methods:**

Cow manure was collected from three local small-scale farmers and composted under controlled conditions, while the bacterial and archaeal communities were determined using shotgun metagenomics at the onset and after 74 days of composting.

**Results:**

The bacterial, archaeal, methanogen, methanotrophs, methylotroph, and nitrifying community structures and their genes were affected by composting for 74 days, but the original composition of these communities determined the changes. Most of these archaeal and bacterial groups showed considerable variation after composting and between the cow manures. However, the differences in the relative abundance of their genes were much smaller compared to those of the archaeal or bacterial groups.

**Discussion:**

It was found that composting of different cow manures did not result in similar bacterial or archaeal communities, and the changes that were found after 74 days were defined by the original populations. However, more research is necessary to determine if other composting conditions will give the same results.

## 1 Introduction

Animal wastes, for example, cow manure, have been applied to arable land to improve soil fertility and increase crop yields (Lee et al., 2023). Apart from providing nutrients for growing crops, that is, most importantly, mineral N, animal wastes improve soil structure and might add plant growth-promoting bacteria (Zhao et al., 2022). Some of these bacteria can liberate phosphorus, produce plant growth-promoting hormones, are antagonistic against the pathogen, fix nitrogen, and/or oxidize ammonium (NH_4_^+^) to nitrate (NO_3_^−^), which is easily taken up by plants as it is not fixed on the soil matrix (Gouda et al., 2018).

However, animal waste can also contain pathogens or opportunistic pathogens for plants, animals, or humans, which might restrict their use in agricultural practices (Blaiotta et al., 2016; Matthews, 2023). The type and quantity of pathogens in the animal waste will depend on the animal, feeding, and characteristics of the manure produced (Abdugheni et al., 2023). Composting can also increase microorganisms that participate in the liberation of plant nutrients (Lucchetta et al., 2023). Additionally, animal manures are an ideal environment for methane (CH_4_) producing archaea. Methanogens thrive under anaerobic conditions in organic material-rich environments and often produce large amounts of CH_4_ in animal wastes (Pampillón-González et al., 2017). According to a United Nations Climate Change Report (2023), the total CH_4_ emissions from livestock manure management was 2.62 kt, with 1.36 kt or 52% from cattle manure. Animal manures might also contain methanotrophs that oxidize CH_4_ under microaerobic conditions (van Bodegom et al., 2001), thereby reducing emissions of this potent greenhouse gas (Nordahl et al., 2023).

Composting is a self-heating, aerobic, and biodegradative process of organic material. The composting process is affected by several characteristics of the organic waste, such as nutrients, pH, moisture content, initial particle size aeration, temperature, and carbon-to-nitrogen (C/N) ratio (Neklyudov et al., 2014; Lin et al., 2022). Composting not only alters the physical and chemical characteristics of the composted organic waste but also alters microbial communities profoundly. For instance, Wan et al. (2021) reported that during composting, the bacterial and fungal diversity decreased, and they also reported large shifts in community composition and species dominance. Composting has also often been applied to animal wastes, such as cow manure, to reduce pathogens (Gurtler et al., 2018) and veterinary antibiotics (Ezzariai et al., 2018), and liberate plant nutrients (Lucchetta et al., 2023). How composting affects the microbial community and their metabolic functioning, that is, their genes, and especially those archaea and bacteria involved in mineral N and methane cycling need further investigation. Therefore, cow manure was collected from three small-scale Mexican farmers. In small-scale farming in central Mexico, cow feces are collected with the maize straw from the stalls in which the animals are kept. This mixture is kept in heaps outside the farms without any further treatment. The composted mixture is spread on agricultural land before the crop-growing season starts.

In this study, cow manure was collected from three different farmers to include variations in cattle farming and its possible effect on the microbial community and its genes. We used cow manure from local farmers for composting as we want to promote the use of compost as a biofertilizer in an area with highly eroded soil and soil low in organic matter content. If the obtained composts are uniform in composition, then they could be used not only as a supplier of nutrients to plants but also as a biofertilizer. The three collected cow manures were composted under controlled conditions, that is, constant water content (WC) and temperature, in the greenhouse for 74 days. The collected cow manure and the composted cow manure were characterized, and the archaeal and bacterial communities were determined through shotgun metagenomics. The aim of this study was to determine how composting affected (i) the archaeal and bacterial community structure (ii) the bacteria and archaea, and the genes involved in mineral N cycling and CH_4_ cycling. It was hypothesized that composting would (i) alter the archaeal and bacterial groups and the genes involved in the N cycle, (ii) favor the nitrifying population and methanotrophs, and (iii) the variation in the bacterial community would be larger than the variation in its genes, that is, metabolic functions. In previous studies based on the 16S rRNA gene, we found that variations in the relative abundance of putative metabolic functions were smaller than those of the bacterial groups (Dendooven et al., 2024). Shotgun metagenomics allows us to determine changes in the relative abundance of genes directly so the last hypothesis can be verified directly. The novelty of this research is that we used shotgun metagenomics to study the effect of composting different organic wastes, that is, cow manure, which allows us to study a wide range of microorganisms and their genes simultaneously.

## 2 Materials and methods

### 2.1 Cow manure collection, characterization, and composting

The local farms selected for sampling cow manure are small, with only a couple of cows. The farmers have some cows, but they have other additional jobs, mostly as laborers. The purpose of the farms is the self-consumption of milk and meat or for selling in the local market. The feed that the cows receive is not standardized or industrialized. The cows are usually fed with crop residues, mainly maize, grass, or other green fodder, depending on the season. The cows do not receive extra pharmacological treatments or nutritional supplements. The cow manure is taken from the cow sheds regularly and kept in piles in the open air without any further treatment. It is collected and spread on farmland before planting starts in the rainy season, that is, June.

Cow manure was collected from three local farmers in the state of Tlaxcala (México). It was obtained from a first farmer in Ixtenco (19°15′00″N, 97°53′00″O), the second was located in Colonia Altamira Guadalupe in the city of Huamantla (19°18′52″N, 97°55′31″O) and the third Ixtacuixtla (19°19′31″N, 98°22′44″O). In each location, three 30 kg subsamples were collected and taken separately to the laboratory. Each of the samples (*n* = 3) from each location (*n* = 3) was characterized, extracted for DNA, and composted separately to avoid pseudoreplication (Heffner et al., 1996).

The experimental design is schematized in Supplementary Figure S1. The water holding capacity (WHC) was determined on each of the cow manure samples (*n* = 3) from the three locations (*n* = 3), and the cow manure was adjusted to 50% WHC, found to be the optimum for C mineralization (Murwira et al., 1990). Three subsamples (*n* = 3) of each cow manure (*n* = 3) were added separately to polyvinyl chloride (PVC) tubes (length 30 cm W 15 cm) closed at the bottom with a PVC end cap with holes so that excess water could drain freely. On 29 April 2022, a 1 kg subsample was taken from each PVC tube (*n* = 9), characterized, and extracted for DNA as described below.

Each PVC tube was covered with fine cloth to keep away insects and placed in a greenhouse. The WC of the cow manure was maintained at 50% WHC by applying distilled water every 2–3 days, depending on the amount of water lost. On 25 May, that is, after 30 days, a 50 g subsample of cow manure was taken from each column, and the WHC was determined. The WC in the cow manure was again adjusted to 50% of the WHC as determined after 30 days. On 12 July, that is, after 74 days of composting, the cow manure was removed from the PVC tubes, characterized, and extracted for DNA as described below.

### 2.2 Characterization of the cow manure

The pH of the cow manure and the composted cow manure was measured in 1:2.5 soil-H_2_O (w:w) suspension using a 716 DMS Titrino pH meter (Metrohm Ltd. CH.-9101 Herisau, Switzerland) fitted with a glass electrode (Thomas, 2018). Total C was determined by oxidation with potassium dichromate and trapping the evolved CO_2_ in NaOH and titrating it with 0.1 M HCl (Amato, 1983), while total N was measured with the Kjeldhal method using concentrated H_2_SO_4_, K_2_SO_4_, and HgO to digest the sample (Bremner, 1996). The CO_2_ emitted after a 7-day incubation was measured by trapping evolved CO_2_ in 1 M NaOH and determined by titration with 0.1 M HCl (Bartha and Pramer, 1965; Jenkinson and Powlson, 1976). The electrolytic conductivity (EC) was determined in a saturated solution extract of the cow manure (Rhoades et al., 1989). The WC of the cow manure and composted cow manure was determined by weighing a fresh 20-g subsample, drying it at 60°C for 24 h, and weighing it again. The WC of each subsample was defined as the difference in weight between the fresh and dried subsample. The WHC of the cow manure and composted cow manure was determined by water saturating a fresh 20 g subsample and leaving it to drain freely overnight. The water-holding content of each subsample was defined as the difference in weight between the water-saturated and drained subsample, and the dried sample. Each characterization was done separately on a subsample from each location (*n* = 3) and each sample (*n* = 3).

### 2.3 DNA extraction

A 0.5-g sub-sample of each cow manure sample (*n* = 9) and composted cow manure (*n* = 9) was extracted for DNA using the QIAGEN kit as stipulated by the provider. Aliquots of 2 mL of each sample were centrifuged at 12,000 g for 5 min, the pellets were resuspended in 200-μL sterile distilled water, 180-μL lysozyme (20 mg/mL) was added to each tube and incubated at 37°C for 1 h. The samples were processed by the QIAcube-robot with the QIAamp DNA Mini (Qiagen, Venlo, The Netherlands) and eluted to a final volume of 200-μL water.

The integrity of DNA was confirmed on 0.8% agarose gel and quantified using Invitrogen’s PicoGreen^®^ dsDNA fluorometric quantitation assay on a NanoDrop^™^ 3300 (Thermo Scientific, Carlsbad, CA) (Supplementary Figure S2). The DNA was sequenced by Centre d’expertise et de services Génome Québec (Montreal, Québec, Canada).

### 2.4 Bioinformatics and assembly

First, raw paired-end reads were mapped against the masked human reference genome GRCh38.p14, and the cow reference genome ARS-UCD1.3 using BBMap^1^ to identify and remove possible human contaminant and cow host sequences as recommended by Bushnell (2014). Unmapped reads to the human genome were quality filtered using Sickle version 1.33 (Joshi and Fass, 2011), that is, paired-end sequences with an average *Q*-score below 20 and shorter than 50 nucleotides were discarded. Unambiguous and unpaired reads that passed quality filtering were kept (-s, singles, or uneven pairs) for assembly. High-quality reads were used as inputs for the MEGAHIT assembler (Li et al., 2015). The multiple *k-*mer lengths option was chosen based on the software developer’s recommendations for large and complex metagenomes [e.g., soil samples (*k*-min 27, *k*-max 127, and *k*-step 10)], and the minimum contig length was set to 500 nt. Open reading frames prediction and protein annotation with assembled contigs were done with Prokka version 1.13 using the *metagenome* setting for highly fragmented datasets^2^ (Seemann, 2014). To quantify the genes, the readings were mapped against the contigs with bowtie2 version 2.5.3 (Langmead and Salzberg, 2012) and quantified with HTSeq version 2.0.3 (Anders et al., 2015).

### 2.5 Metabolic pathways

Metabolic pathways prediction was done as follows. The generic feature annotation file (GFF Prokka’s annotation output) was filtered to retain all genes with a KO identifier assigned. The “Minimal set of Pathways” tool (MinPath version 1.4, Ye and Doak, 2009) was used to obtain a conservative estimation of pathways to visually them on the microbial metabolic map through the interactive pathway explorer web-based tool (iPath version 3, https://pathways.embl.de/).

### 2.6 Nitrogen cycle-related genes

The ORF prediction of assembled contigs was done with prodigal version 2.6.3 (Hyatt et al., 2010). Sequences were aligned against the NCyc database (Tu et al., 2019) using DIAMOND aligner with an *e*-value of 1 × 10^−5^ and 80% of identity (Buchfink et al., 2015). The NCyc database was constructed with nitrogen cycle gene families from UniProt and from different orthology databases [clusters of orthologous genes (COG), SEED, Kyoto Encyclopedia of Genes and Genomes (KEGG), and evolutionary gene genealogy Non-supervised Orthologous Groups (eggNOG)]^3^ (Tu et al., 2019).

### 2.7 Microbial community analysis

Fastq raw files were cleaned and trimmed with trimmomatic for the diversity analysis. Contigs were assembled with SPADES in meta configuration, followed by annotation with kraken2 using Genbank RefSeq database (accessed in July 2023). Only archaeal and bacterial results were used for microbial community analysis.

### 2.8 Statistical analysis

All statistical analyses were done in R version 4.2.2 (R Core Team, 2019) within the RStudio environment (Version 2023.09.0 + 463). Alpha diversity of the soil bacterial community was determined based on the Hill numbers at different *q* orders (*q* = 0, 1, and 2) (Chao et al., 2010). The Hill number at *q* = 0 gives the species richness, *q* = 1 is the Shannon entropy and denotes frequently occurring species, and *q* = 2 is the inverse Simpson and characterizes dominant species (Chao et al., 2014) and they were calculated with the HillR package version 0.5.1 (Li, 2021). A non-parametric analysis (t1way test of the WRS2 package, version 1.1-0, Mair and Wilcox, 2020) was used to determine the effect of the origin of the cow manure and composting on the Hill numbers. The changes in the bacterial community due to composting were determined with the betapart R package (Baselga and Orme, 2012).

Ordination [principal component analysis (PCA)] and multivariate comparison (PERMANOVA) were made with converted sequence data using the centered log-ratio transformation test returned by the aldex.clr argument ALDEx2 (version 1.21.1) (Gloor et al., 2020). The FactoMineR (version 2.3) package (Husson et al., 2020) was used for the PCA and the vegan (version 2.5-6) package to determine the homogeneity of groups dispersions (dispersion within the cow manure and composted cow manure) (Anderson, 2017; Oksanen et al., 2019). The Mantel test for dissimilatory matrices was used to calculate the Mantel statistic (*r*) and the *p*-value, while a constrained and unconstrained redundancy analysis (RDA) was done with the unconstrained and constrained characteristics of the cow manure with different archaeal and bacterial groups, and genes with the vegan (version 2.5-6) package (Anderson, 2017; Oksanen et al., 2019). The characteristics of the cow manure and composted cow manure that were included in the constrained RDA were those that had a significant effect on the bacterial or archaeal groups or genes, as indicated by the Mantel test (*p* < 0.05). None of the characteristics of the cow manure and composted cow manure was significantly correlated with the nitrite-oxidizing bacteria (NOB), so the constrained RDA was done with all characteristics except pH and total N as they were never correlated significantly with any bacterial or archaeal group. The ordistep function (based on *p*-values) in the RDA of the vegan package was used to develop a model by permutation tests.

The effect size, which is defined as the difference between groups divided by the maximum dispersion within group A or B, was calculated after a centered log-ratio transformation with the aldex.ttest argument [ALDEx2 (version 1.18), Gloor et al., 2020]. The effect size was calculated by comparing the archaeal and bacterial groups and genes in the cow manure at the onset of the experiment and after 74 days of composting. Only effect sizes that were large were considered (≤ −0.8 or ≥0.8) (Kim, 2015). The effect size was plotted vs. the expected *p*-value of the Kruskal–Wallis test in Microsoft^®^ Excel for Mac version 16.62. The variance of relative abundance of the bacterial and archaeal species and the genes were calculated for the three compost samples from each of the three locations, and the variance between the three locations.

### 2.9 Sequence database deposition

The raw sequences dataset was deposited in the NCBI-SRA (Sequence Read Archive) under BioProject SUB14117382 accession number PRJNA770100.

## 3 Results

### 3.1 Effect of composting on cow manure characteristics

**Figure 1:**
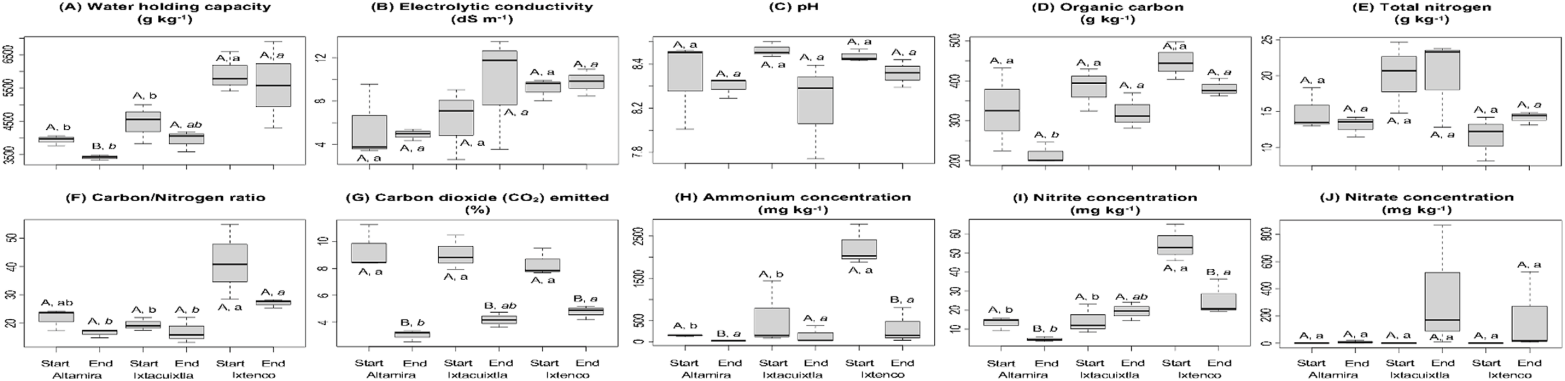
Box plots with the characteristics of the compost, that is, **(A)** water-holding capacity, **(B)** electrolytic conductivity (EC), **(C)** pH, **(D)** organic carbon, **(E)** total nitrogen (TN), **(F)** carbon/nitrogen ratio, **(G)** carbon dioxide (CO_2_) emitted, **(H)** ammonium concentration, **(I)** nitrite concentration, and **(J)** nitrate concentration, from three locations at the onset of the experiment (Start) and after 74 days of composting (End). Samples with the same capital letter are not affected by composting, that is, comparison between the cow manure and the composted cow manure for each location, samples with the same letter are cow manures from the different locations that are not significantly different, that is, comparison of the cow manures, while values with the same letter in italic are composted cow manures from the different locations that are not significantly different, that is, comparison of the composted cow manures, (*p* < 0.05).

The WHC in the cow manure ranged from 3,935 to 5,930 g kg^−1^ and was significantly higher in the cow manure from Ixtenco than in the cow manure from Altamira and Ixtacuixtla (*p* < 0.05) (Figure 1A). Composting did significantly reduce the WHC in the cow manure from Altamira but not in that from Ixtenco and Ixtacuixtla (*p* < 0.05). The EC in the cow manure ranged from 5.6 to 9.2 dS m^−1^, and in the composted cow manure, from 4.9 to 9.8 dS m^−1^ (Figure 1B). The EC was similar in the cow manures and in the composted cow manures, and composting did not affect the EC significantly. The pH in the cow manure ranged from 8.5 to 8.7, and in the composted cow manure, from 8.2 to 8.5 (Figure 1C). The pH was similar in the cow manures and in the composted cow manures, and composting did not affect the pH significantly. The organic C in the cow manure ranged from 327 to 449 g kg^−1^, and in the composted cow manure, from 216 to 382 g kg^−1^ (Figure 1D). The organic C was similar in the cow manures but significantly lower in the composted cow manure from Altamira than in that from Ixtenco and Ixtacuixtla (*p* < 0.05). Composting did not affect the organic C significantly. The total N ranged from 11.5 to 20.1 g kg^−1^ in the cow manures and between 13.1 and 20.0 g kg^−1^ in the composted cow manures (Figure 1E). Total N was similar in the cow manures and composted cow manure, and composting did not affect the total N significantly. The C/N ratio ranged from 19.1 to 39.0 in the cow manures and between 16.1 and 27.0 in the composted cow manures (Figure 1F). The total C/N ratio was similar in the cow manures and composted cow manure, and composting did not affect the C/N ratio significantly. The CO_2_ emitted from the cow manure in a 7-day incubation ranged from 8.4 to 9.4% of the organic-C at the onset of the experiment and from 3.0 to 4.7% after composting the cow manure (Figure 1G). The CO_2_ emitted in a 7-day incubation was not different between the cow manures, but after composting, it was significantly larger from the Ixtenco composted cow manure than from the Altamira one (*p* < 0.05). Composting reduced the CO_2_ emitted in a 7-day incubation in each of the studied cow manures (*p* < 0.05). The NH_4_^+^ concentration in the cow manures ranged from 147 to 2,233 mg N kg^−1^ and from 27 to 332 mg N kg^−1^ in the composted cow manures (Figure 1H). Composting reduced the NH_4_^+^ concentration in cow manures from Altamira and Ixtenco, but not from Ixtacuixtla (*p* < 0.05). The NH_4_^+^ concentration in the cow manure from Ixtenco was significantly larger than from Altamira and Ixtacuixtla (*p* < 0.05), but after composting, it was similar. The NO_2_ concentration in the cow manures ranged from 13 to 55 mg N kg^−1^ and from 5 to 332 mg N kg^−1^ in the composted cow manures (Figure 1I). Composting reduced the NO_2_ concentration in the cow manures from Altamira and Ixtenco, but not from Ixtacuixtla (*p* < 0.05). The NO_2_ concentration in the cow manure from Ixtenco was significantly larger than that from Altamira and Ixtacuixtla (*p* < 0.05). After composting, the NO_2_ concentration in the cow manure from Ixtenco was significantly larger than from Altamira (*p* < 0.05). No NO_3_^−^ was detected in the cow manures but ranged from 9 to 349 mg N kg^−1^ in the composted cow manures (Figure 1J). The NO_2_^−^ concentration in the cow manure from Ixtenco was significantly larger than that from Altamira and Ixtacuixtla (*p* < 0.05). After composting, the NO_3_^−^ concentration in the cow manures was similar (*p* < 0.05).

### 3.2 Bacteria

#### 3.2.1 The bacterial community structure

Overall, 66,252,686 bacterial sequences were obtained that included 39 phyla, 2,931 genera, and 9,152 species. The α diversity was not affected by composting, but significantly different between the cow manures (considering the onset and after 74 days of composting) at Hill numbers *q* = 1 (frequent species) and *q* = 2 (dominant species) (Supplementary Figure S3A). The dissimilarity index for bacterial species was small after 74 days of composting (0.063) and most changes were due to 1-to-1 substitution (0.050).

**Figure 2:**
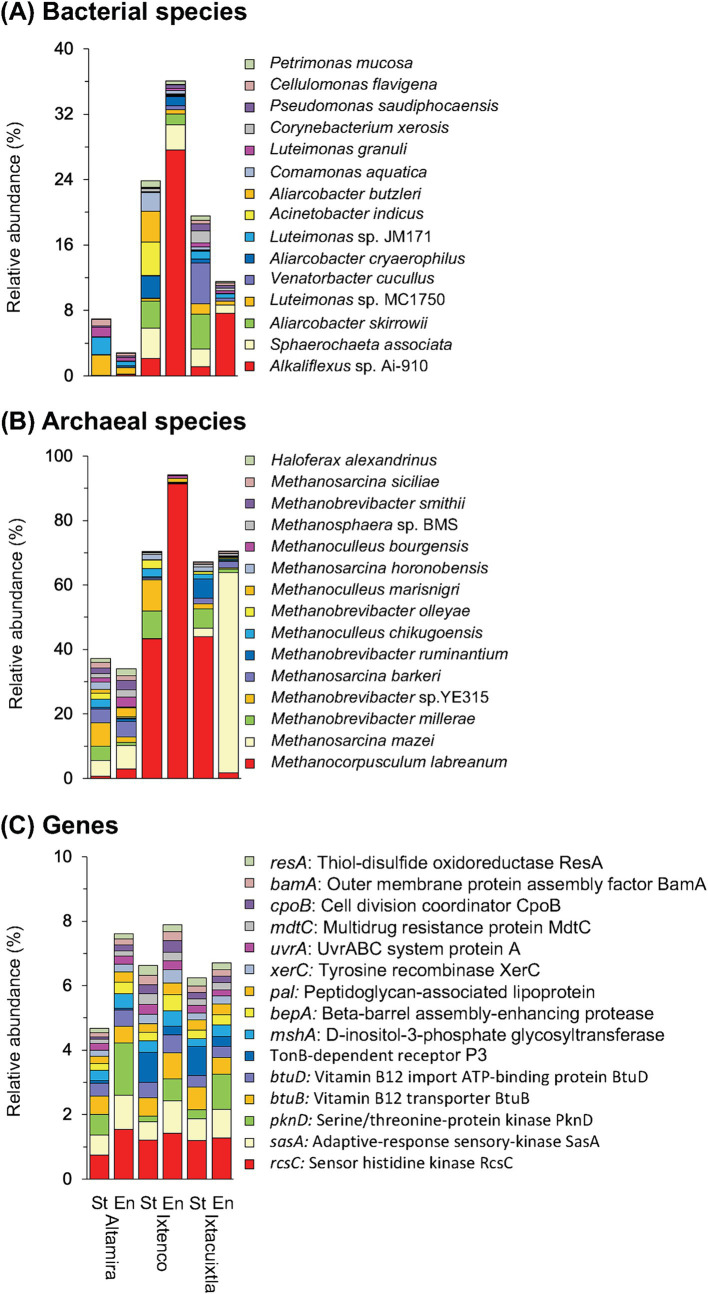
Barplots with the relative abundance (%) of the 15 most abundant **(A)** bacterial species, **(B)** archaeal species, and **(C)** genes in the cow manures from three locations at the start of the experiment (St) and after 74 days composting (En).

**Figure 3:**
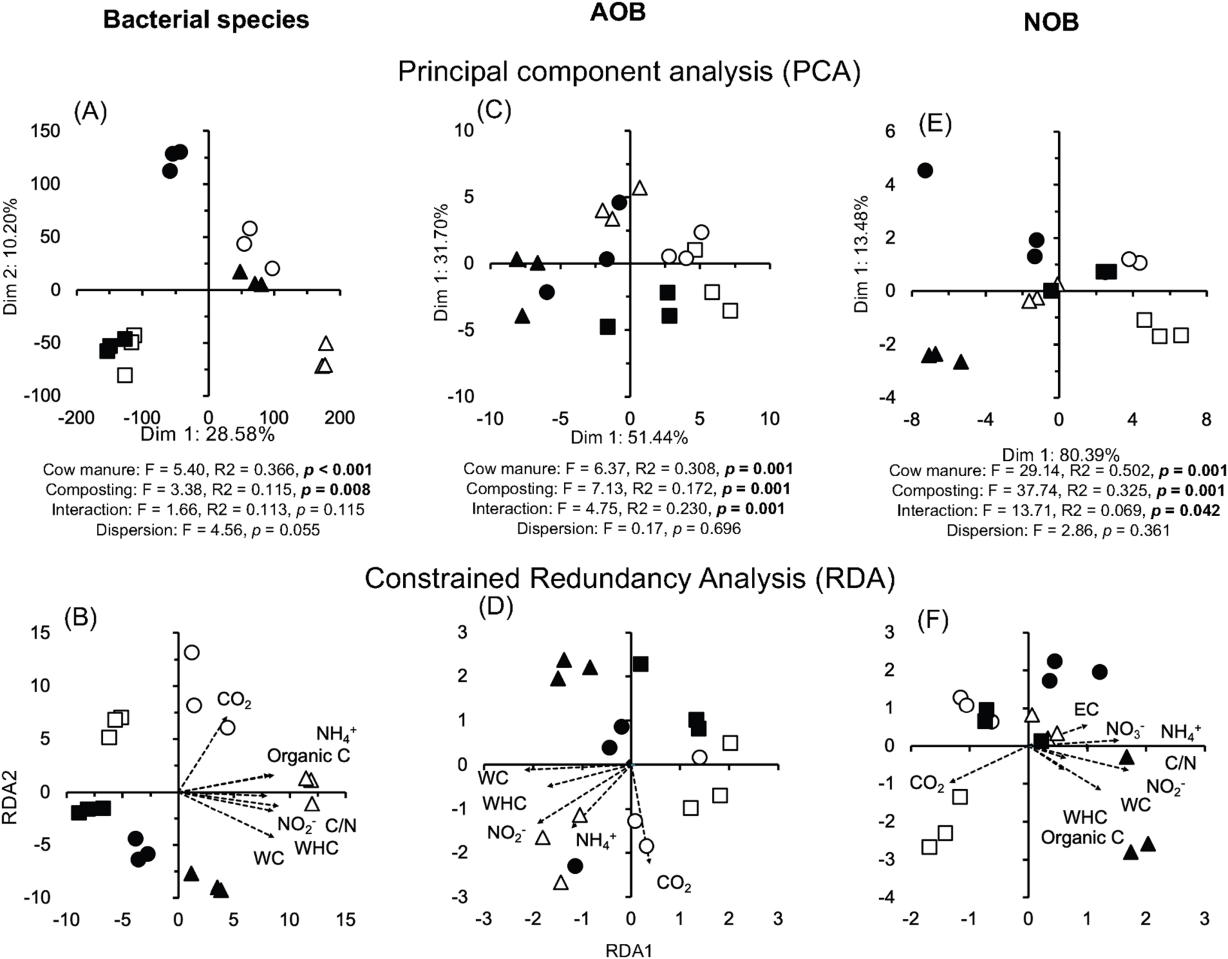
**(A)** Principal component analysis (PCA) and **(B)** constrained redundancy analysis (RDA) with all bacterial species, **(C)** PCA and **(D)** RDA with the ammonium oxidizing bacteria (AOB), and **(E)** PCA and **(F)** RDA with the nitrite-oxidizing bacteria (NOB) in the cow manure from Altamira at the beginning of the experiment (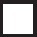) and after 74 days composting (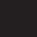), from Ixtacuixtla at the beginning (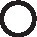) and after 74 days composting (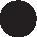), and from Ixtenco at the beginning (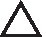) and after 74 days composting (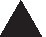). The results of a permutational multivariate analysis of variance (PERMANOVA) test comparing the community structure in the different cow manures, composted cow manures, and their interaction is given with *p*-values in bold, indicating that differences between the cow manures or composted cow manures were significant at <0.05 and the permutation multivariate analysis of dispersion (PERMDISP) test was used to determine the homogeneity of groups dispersions (dispersion within cow manures vs. dispersion between composted cow manures) (Anderson, 2006).

Pseudomonadota (formerly, Proteobacteria) dominated in the cow manure and the compost (mean relative abundance of all samples 59.94%), while members of the Actinomycetota were the second most abundant phyla in one of them and Bacteroidota in the other two (Supplementary Figure S4A). Composting enriched some bacterial phyla, for example, Bacteroidota, and decreased the relative abundance of others, for example, Bacillota (formerly, Firmicutes), but the effect was sometimes determined by the location where the cow manure was collected. This effect was even more outspoken considering bacterial genera and species (Figure 2A; Supplementary Figure S4B). Despite this, the effect size (or the difference between groups divided by the maximum dispersion within group A or B) of the relative abundance of many bacterial genera and species had a very large effect size ≤ −1.4 or ≥1.4 and highly significant (*p* < 0.001) (Supplementary Figure S5A). The PCA separated the bacterial communities in different cow manures and the composted cow manure (Figure 3A). Consequently, cow manure and composting had a significant effect on the bacterial community structure considering all bacterial species (*p* < 0.05). The Mantel test indicated a significant effect of WHC, organic C, CO_2_ emitted in 7 days, C/N ratio, and NH_4_^+^ and NO_2_^−^ content on the bacterial community (*p* < 0.05) (Supplementary Table S1). The constrained RDA, considering the characteristics that had a significant effect on the bacterial community, clearly separated the different cow manures and the composted cow manures (Figure 3B). The ordistep function in RDA resulted in a model that included organic material (*p* = 0.05) and NO_2_^−^ (*p* = 0.01).

#### 3.2.2 Ammonium and nitrite-oxidizing bacteria

**Figure 4:**
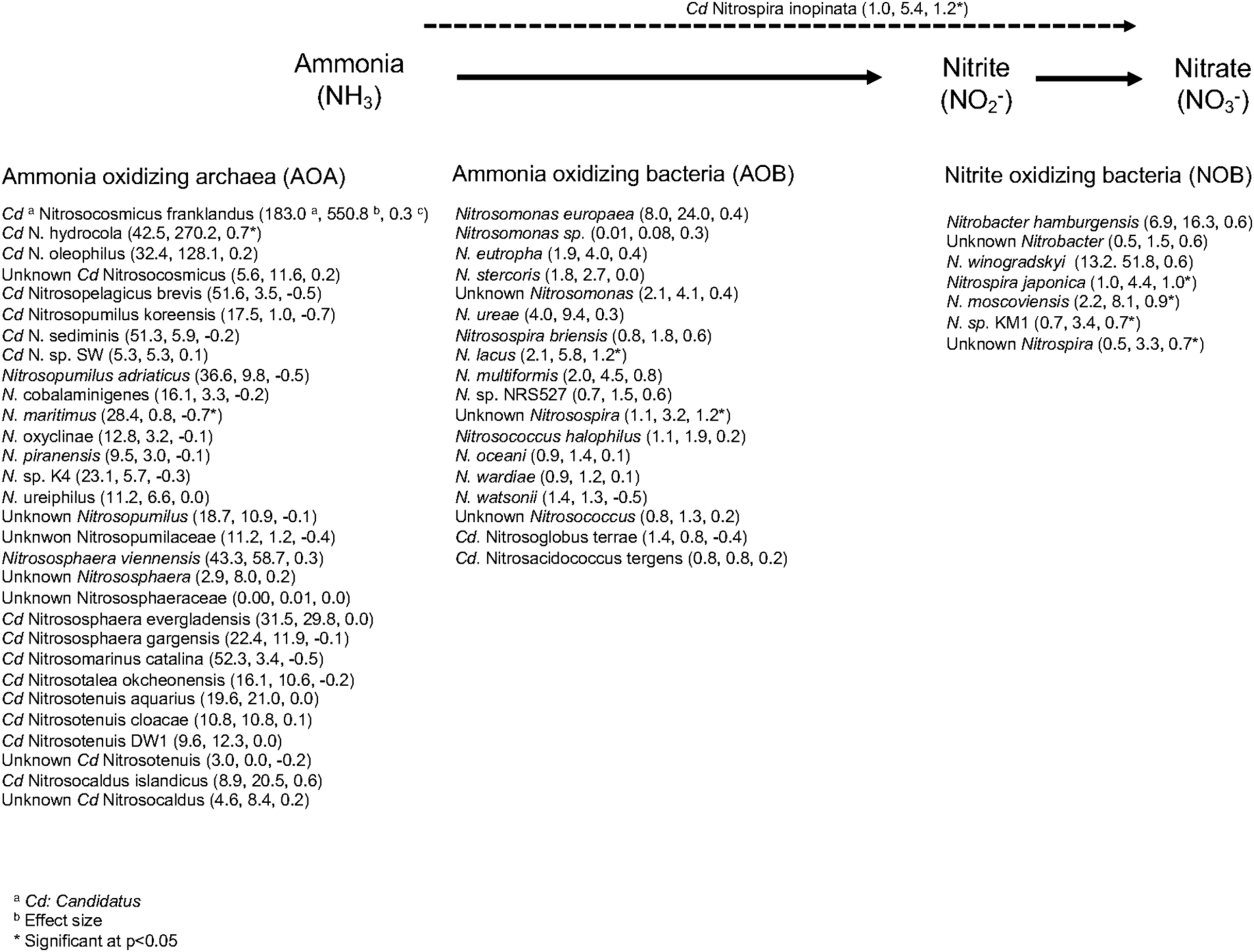
List of ammonium-oxidizing bacteria (AOB), ammonium-oxidizing archaea (AOA), and nitrite-oxidizing bacteria (NOB) found in the different cow manures and composting cow manures. The values between parenthesis are ^a^the relative abundance (×10^−3^) in the cow manure at the onset of the experiment, ^b^the relative abundance (×10^−3^) in the composted cow manure after 74 days of composting and ^c^the effect size, which is defined as the difference between groups divided by the maximum dispersion within group A or B, was calculated with the ALDEx2 package using the aldex.ttest argument. A negative value indicates that the relative abundance of the nitrifier was higher in the cow manures than in the composted cow manures, and a positive value indicates opposite.

A wide range of ammonium oxidizing bacteria (AOB) were detected in the cow manure, but the diversity of NOB was low (Figure 4). *Nitrosomonas europaea* was the most abundant AOB, while *Nitrobacter winogradskyi* was the most abundant nitrite oxidizer. Only one member of the comammox community, that is, complete ammonia oxidizers, was detected, that is, *Cd. Nitrospira inopinata* in the cow manure (1.0 × 10^−3^) and composted cow manure (5.4 × 10^−3^). The relative abundance of most AOB, for example, *N. europaea* 3.0 times, and all NOB, for example, *N. winogradskyi* 3.9 times, increased sharply after 74 days of composting. The PCA separated the different cow manures and the composted cow manures considering the AOB (Figure 3C). The effect of composting and cow manures on the AOB was significant, but not the dispersion (*p* < 0.05). The Mantel test indicated a significant positive correlation of WC, WHC, CO_2_ emitted in 7 days, and the NH_4_^+^ and NO_2_^−^ content on the AOB (*p* < 0.05) (Supplementary Table S1), but no characteristic was significantly correlated to the NOB. The constrained RDA considering the characteristics that had a significant effect on the AOB, clearly separated the different cow manures and the composted cow manures (Figure 3D). The ordistep function in RDA resulted in a model that included the CO_2_ emitted in 7 days (*p* = 0.030) and WC (*p* = 0.005) for AOB and NOB (*p* = 0.015 and *p* = 0.005, respectively).

#### 3.2.3 Methylotrophs and methanotrophs

**Figure 5:**
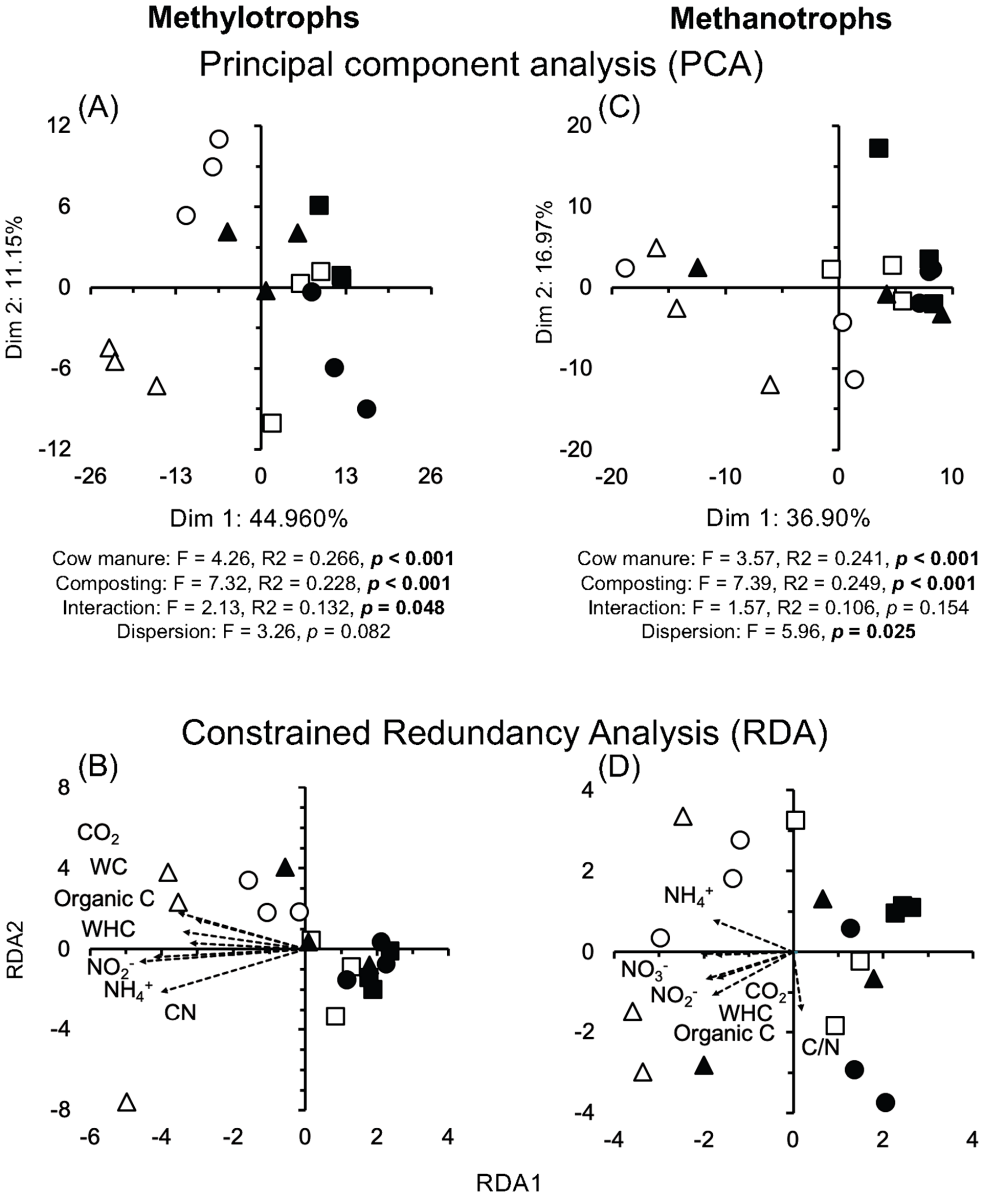
**(A)** Principal component analysis (PCA) and **(B)** constrained redundancy analysis (RDA) with methylotrophs, and **(C)** PCA and **(D)** RDA with methanotrophs in the cow manure from Altamira at the beginning of the experiment (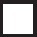) and after 74 days composting (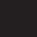), from Ixtacuixtla at the beginning (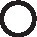) and after 74 days composting (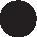), and from Ixtenco at the beginning (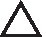) and after 74 days composting (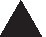). Explanations of the PERMANOVA and PERMDISP analysis are given in the legend to Figure 3.

Most methylotrophs were enriched after composting the cow manure for 74 days. The effect of composting and cow manures on the methylotrophs was highly significant, but the interaction was also significant (*p* < 0.05) (Figure 5A). The dispersion was not significant. The Mantel test indicated a significant effect of WHC, organic C, CO_2_ emitted in 7 days, C/N ratio, and NH_4_^+^ and NO_2_^−^ content on the methylotrophs (*p* < 0.05) (Supplementary Table S1). The constrained RDA, considering the characteristics that had a significant effect on the methylotrophs, separated the different cow manures and the composted cow manures (Figure 5B).

All aerobic obligate methanotrophs being obligate, for example, *Methylotuvimicrobium alcaliphilum*, or facultative, were enriched after composting the cow manure for 74 days. The PCA clearly separated the different cow manures and the composted cow manures considering methylotrophs (Figure 5C). The variation in the cow manures was larger considering methanotrophs, but the composted cow manures were grouped. The effect of composting and cow manures on the methanotrophs was highly significant, but the dispersion was also significant (*p* < 0.05). The Mantel test indicated a significant effect of WHC, organic C, CO_2_ emitted in 7 days, C/N ratio, and NH_4_^+^, NO_2_^−^ and NO_3_^−^ content on the methanotrophs (*p* < 0.05) (Supplementary Table S1). The constrained RDA, considering the characteristics that had a significant effect on the methanotrophs, separated the different cow manures and the composted cow manures (Figure 5D). The ordistep function in RDA resulted in a model that included only the NH_4_^+^ concentration (*p* = 0.005) for both methylotrophs and methanotrophs.

### 3.3 Archaea

#### 3.3.1 The archaeal community structure

Overall, 446,000 archaeal sequences were obtained that included 9 phyla, 235 genera, and 479 species. The archaeal alpha diversity was not affected by composting but was significantly different between the cow manures (considering the onset and after 74 days of composting) at *q* = 0, 1, and 2 (*p* < 0.05) (Supplementary Figure S3B). The dissimilarity index for archaeal species was small (0.269) but larger than that of bacteria after 74 days of composting, and most changes were due to 1-to-1 substitution (0.236).

**Figure 6:**
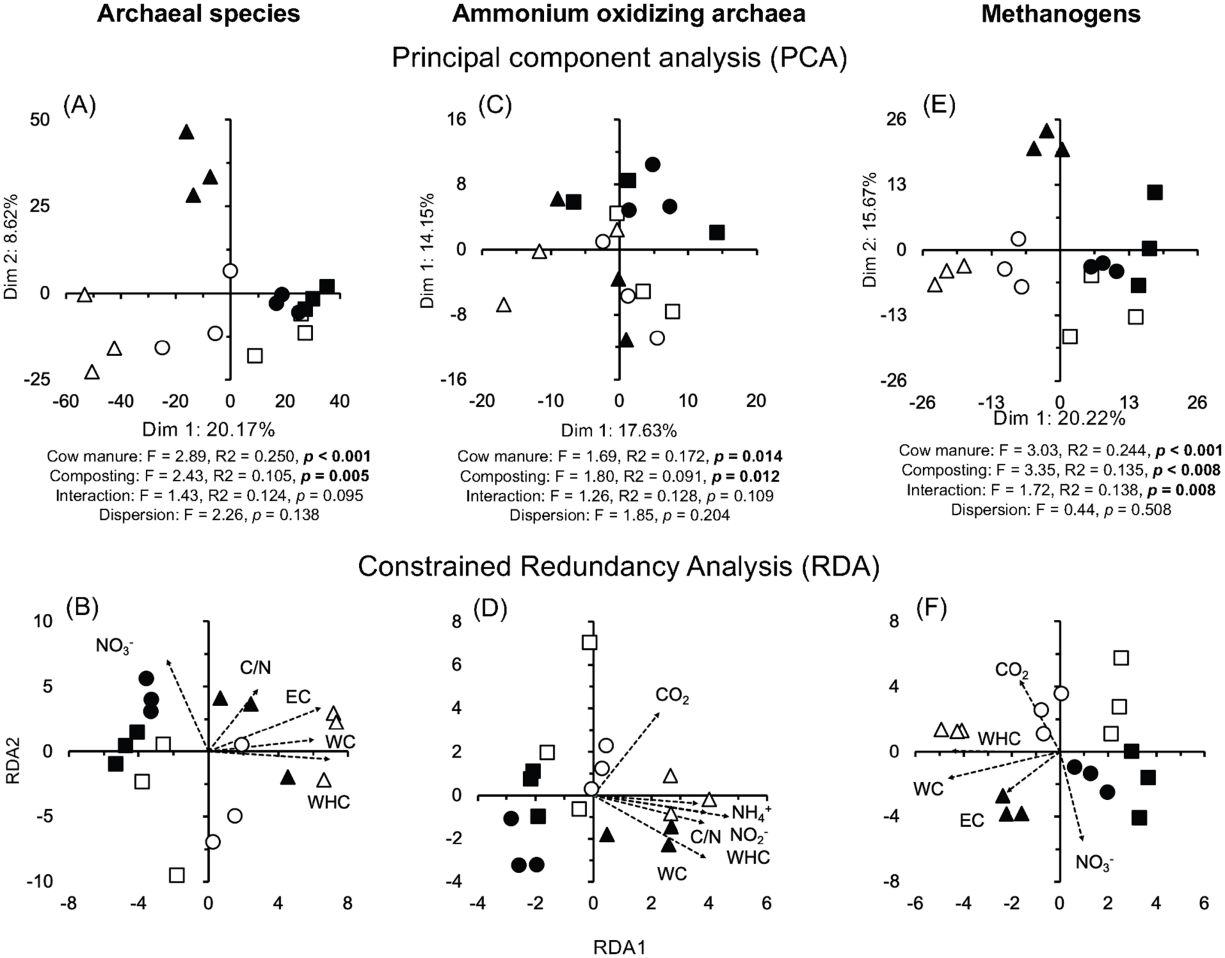
**(A)** Principal component analysis (PCA) and **(B)** constrained redundancy analysis (RDA) with all archaeal species, **(C)** PCA and **(D)** RDA with the ammonium oxidizing archaea (AOA), and **(E)** PCA and **(F)** RDA with the methanogens in the cow manure from Altamira at the beginning of the experiment (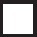) and after 74 days composting (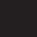), from Ixtacuixtla at the beginning (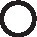) and after 74 days composting (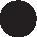), and from Ixtenco at the beginning (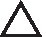) after 74 days composting (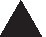). Explanations of the PERMANOVA and PERMDISP analysis are given in the legend to Figure 3.

Members of nine different archaeal phyla were detected and Euryarchaeota dominated in the cow manure and its compost (97.55%) (Supplementary Figure S4B). Composting enriched some archaeal genera, for example, *Methanocorpusculum*, and species *Methanocorpusculum labreanum*, and decreased the relative abundance of others, for example, *Methanobrevibacter millerae*, but the effect was sometimes determined by the origin of the cow manure, for example, *Methanosarcina mazei* (Figure 2B; Supplementary Figure S4D). Despite this, composting had a large (effect size ≤ −1.4 or ≥1.4) and highly significant effect on the relative abundance of some archaeal species (*p* < 0.001) (Supplementary Figure S5B). The PCA clearly separated the archaeal communities in different cow manures, but not always in the composted cow manure (Figure 6A). Composting changed the archaeal community in two of the three cow manures but not so clearly in the third. Consequently, cow manure and composting had a significant effect on the archaeal community structure considering all archaeal species (*p* < 0.05). The Mantel test indicated a significant effect of EC, WHC, C/N ratio, and NO_3_^−^ content on the archaeal community (*p* < 0.05) (Supplementary Table S1). The constrained RDA, considering the characteristics that had a significant effect on the archaeal community, clearly separated the different cow manures and the composted cow manures (Figure 6B). The ordistep function in RDA for archaeal species resulted in a model that included only WHC (*p* = 0.005).

#### 3.3.2 Ammonium oxidizing archaea

A wide range of ammonium oxidizing archaea (AOA) were detected in the cow manure (Figure 4). *Cd. Nitrosocosmicus franklandus* was the most abundant AOA. The relative abundance of some AOA increased sharply after 74 days of composting, but it also decreased for others. The PCA separated the different cow manures and the composted cow manures considering AOA (Figure 6C). The effect of composting and cow manures on the AOA was significant, but not the dispersion (*p* < 0.05). The Mantel test indicated a significant positive correlation between the AOA and the WC, WHC, CO_2_ emitted in 7 days, C/N ratio, and NH_4_^+^ and NO_2_^−^ content (*p* < 0.05) (Supplementary Table S1). The constrained RDA considering the characteristics that had a significant effect on the AOA, clearly separated the different cow manures and the composted cow manures (Figure 6D). The ordistep function in RDA for AOA resulted in a model that included WHC (*p* = 0.005) and the CO_2_ emitted in 7 days (*p* = 0.045).

#### 3.3.3 Methanogens

Different archaeal orders that include methanogenic species were detected in the cow manure. Methanosarcinales, the most abundant archaeal order in the cow manure was replaced by Methanomicrobiales as the most abundant archaeal group after 74 days of composting. Members of Methanobacteriales, Methanonatronarchaeales, and Methanococcales were also enriched after composting, but the relative abundance of Methanomassiliicoccales and Methanocellales decreased. The PCA clearly separated the different cow manures and the composted cow manures, although with some large variation between the samples collected at the same location (Figure 6E). The effect of composting and cow manures on the methanogens was highly significant, but the interaction was also highly significant (*p* < 0.01). The dispersion was not significant. The Mantel test indicated a significant effect of EC, WHC, CO_2_ emitted in 7 days, C/N ratio, and NO_3_^−^ content on the methanogens (*p* < 0.05) (Supplementary Table S1). The constrained RDA considering the characteristics that had a significant effect on the methanogens, clearly separated the different cow manures and the composted cow manures, although with some large variation between the samples collected at the same location (Figure 6F). The ordistep function in RDA for methanogens resulted in a model that included WHC (*p* = 0.005) and the CO_2_ emitted in 7 days (*p* = 0.005).

### 3.4 Genes

#### 3.4.1 The overall gene structure

In total, 127,640,255 sequences were obtained related to 11,645 genes. The Hill number of the genes at *q* = 2 was affected significantly by composting, and the Hill number at *q* = 1 was significantly different between the cow manures (considering the onset and after 74 days of composting) (*p* < 0.05) (Supplementary Figure S3C). The dissimilarity index for genes was small (0.261) and similar to that of archaea after 74 days of composting, and most changes were due to 1-to-1 substitution (0.181), although some losses of genes also occurred, that is, they were not detected.

**Figure 7:**
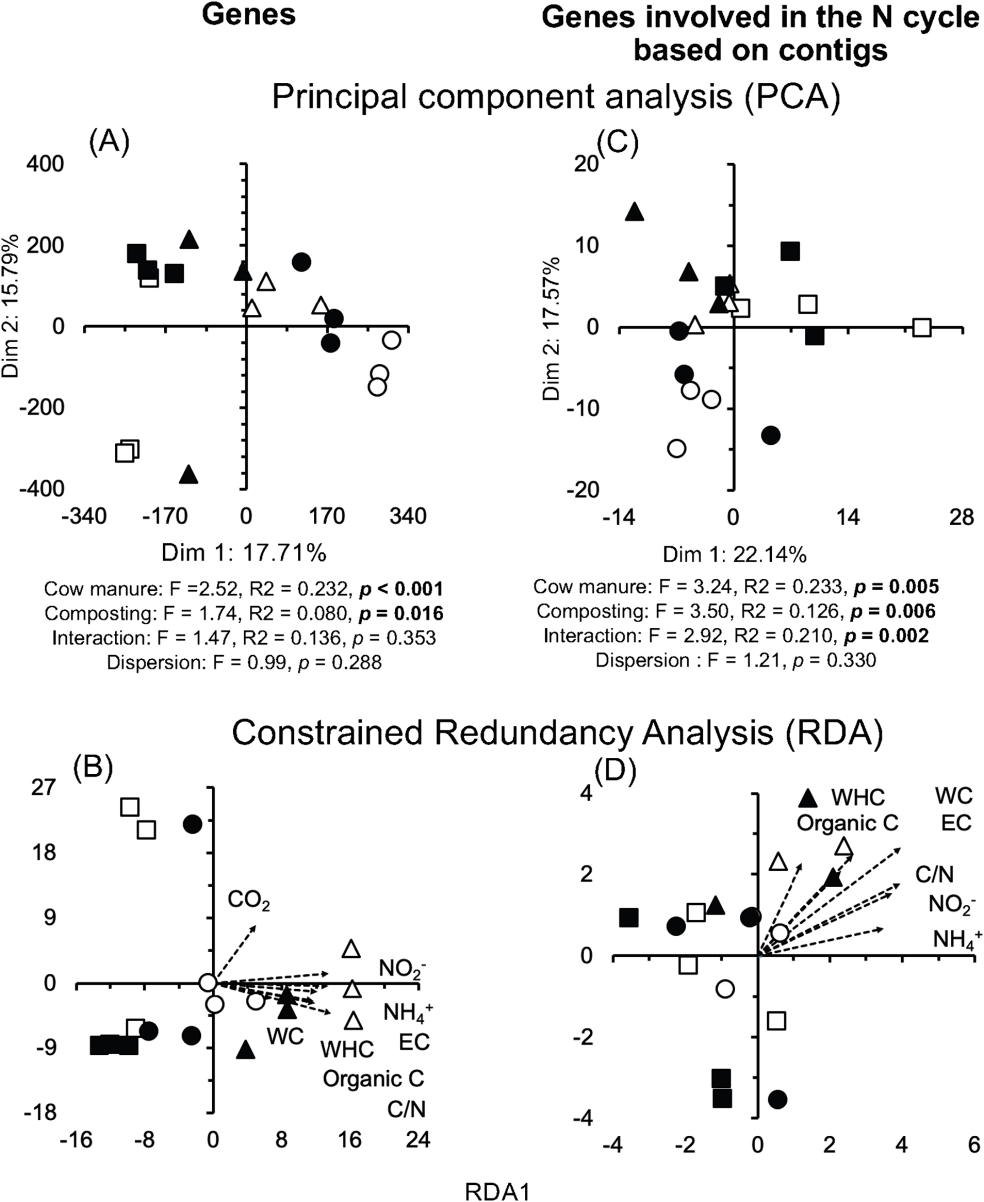
**(A)** Principal component analysis (PCA) and **(B)** constrained redundancy analysis (RDA) with the sequences of all genes, and **(C)** PCA and **(D)** RDA with the genes involved in the N cycle in the cow manure from Altamira at the beginning of the experiment (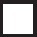) and after 74 days composting (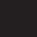), from Ixtacuixtla at the beginning (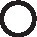) and after 74 days composting (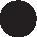), and from Ixtenco at the beginning (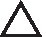) after 74 days composting (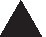). Explanations of the PERMANOVA and PERMDISP analysis are given in the legend to Figure 3.

The *rcsC* (sensor histidine kinase RcsC, 1.23%) and *sasA* gene (adaptive-response sensory-kinase SasA, 0.81%) were the most abundant genes (Figure 2C). Composting had a large (effect size ≤ −1.4 or ≥1.4) and highly significant effect on the relative abundance of many genes (*p* < 0.001) (Supplementary Figure S5C). The PCA clearly separated the gene structure in different cow manures and the composted cow manures, although with some large variation between the samples collected at the same location (Figure 7A). Consequently, cow manure and composting had a significant effect on the gene structure (*p* < 0.05). The Mantel test indicated a significant effect of EC, WHC, organic C, and CO_2_ emitted in 7 days, C/N ratio, and NH_4_^+^ and NO_2_^−^ content on the genes (*p* < 0.05) (Supplementary Table S1). The constrained RDA considering the characteristics that had a significant effect on the genes, separated the different cow manures and the composted cow manures, although with some large variation between the samples collected at the same location (Figure 7B). The ordistep function in RDA for all genes resulted in a model that included WC (*p* = 0.005) and the CO_2_ emitted in 7 days (*p* = 0.025).

#### 3.4.2 The genes involved in the N cycle

Many genes involved in the N cycle were detected in the cow manure and the composted cow manure (Supplementary Table S2). The relative abundance of more genes increased after 74 days of composting than those that showed a decrease (*p* < 0.05) (Supplementary Table S2). The PCA separated the different cow manures and the composted cow manures considering the genes involved in the N cycle, although with some large variation between the samples collected at the same location (Figure 7C). The Mantel test indicated a significant effect of EC, WHC, organic C, C/N ratio, and NH_4_^+^ and NO_2_^−^ content on the genes involved in the N cycle (*p* < 0.05) (Supplementary Table S1). The constrained RDA considering the characteristics that had a significant effect on the genes involved in the N cycle, separated the different cow manures and the composted cow manures, although with some large variation between the samples collected at the same location (Figure 7D). The ordistep function in RDA for the genes involved in the N cycle resulted in a model that included only WHC (*p* = 0.015).

### 3.5 Comparison between the metabolic functions and the archaeal and bacterial communities

The variations in relative abundances within each of the sampled compost (*n* = 3) at the onset of the experiment and after 74 days of composting were largest for the archaeal species (mean 116 × 10^−3^) and lowest for the genes (9 × 10^−6^) (Supplementary Figure S6). For instance, the maximum variation for archaeal species was 1,200, that is, *M. labreanum* in the cow manure from Ixtacuixtla at the onset of the experiment, but only 1.35 for the *pknD* gene in Ixtacuixtla after composting for 74 days. A similar pattern emerged when comparing the variation in the three cow manures sampled and after 74 days of composting (Supplementary Figure S7). For instance, the maximum variation for archaeal species was 2,617, that is, *M. labreanum* after composting the cow manure for 74 days, but only 0.26 for the TonB-dependent receptor P3. A much larger variation was also detected in the ratio between the relative abundance of bacterial or archaeal groups at the onset of the experiment and after 74 days of composting compared to the changes in the relative abundance of the genes (Supplementary Figure S8).

## 4 Discussion

### 4.1 Compost characteristics

The characteristics of cattle manure are defined by waste management, type of animal, and feeding (Aboudi et al., 2021). As such, characteristics of different cow manures are highly variable, but they generally contain large amounts of organic material (Shepherd et al., 2002). In this study, the cow manures were slightly alkaline with a pH of 8.6 which is near the optimum for composting, as reported by Ge et al. (2022). However, they had a high EC 7.0 dS m^−1^ (mean of the three samples), which might limit their repeated application to arable soil. The organic C content was high and ranged between 327 and 449 g C kg^−1^ with a low C/N ratio except in the cow manure from Ixtenco. A C/N ratio in animal waste of 25–30 should allow for adequate composting, although a lower C/N ratio (15–20), as found in two of the three composts, might facilitate the composting process (Guo et al., 2012). The NH_4_^+^ and NO_2_^−^ content was highly variable in the cow manure as reported often (e.g., Risberg et al., 2017). The detection of NO_2_^−^ suggested that oxidation of NH_4_^+^ occurred (nitrification), so NO_3_^−^ should have been formed, but none was detected, so it must have been reduced (Shi et al., 2017). As such, aerobic microsites coexisted with anaerobic ones, which occurs even in well-aerated manures when the oxygen consumed by the heterotrophic microorganisms matches the oxygen flux in parts of the composting cow manures (Lv et al., 2020).

Cow manure contains large amounts of organic C content that is easily mineralized, but once it is mineralized, only more resistant C substrates remain, which are more slowly decomposed by the microorganisms (Miranda-Carrazco et al., 2022). In this study, 8.9% of the organic C was emitted as CO_2_ in a 7-day aerobic incubation (mean of the three samples) at the onset of the experiment, but more than halved after 74 days of composting which indicated that most easily decomposable organic material was mineralized (Inbar et al., 1993). The C/N ratio also dropped after composting, which indicated that a mature product was obtained after 74 days (Goyal et al., 2005).

### 4.2 Bacteria

#### 4.2.1 The bacterial community structure

Different bacterial species dominated in the cow manures, that is, *Luteimonas* sp. MC1750, *Venatorbacter cucullus* or *Acinetobacter indicus*. These bacterial species have been found before in cattle waste. Members of *Luteimonas* participated in lignocellulose degradation during the composting of cattle manure and straw, as the application of mannooligosaccharides enriched them (Zhang H. et al., 2022; Zhang Y. et al., 2022). Saeedi et al. (2021) isolated the Gram-negative, aerobic, halotolerant, motile, rod-shaped, and predatory bacterium *V. cucullus* recently from a bovine slurry tank using *Campylobacter hyointestinalis* as prey. *A. indicus* strain ZJB20129 isolated from an urban sewage treatment showed the capacity of heterotrophic nitrification-aerobic denitrification (Ke et al., 2022), but the species is also an opportunistic pathogen (Klotz et al., 2017).

Changes in alpha diversity of the bacterial species after composting are determined by the characteristics of the cow manures, for example, pH, N content and composition of the organic material, and the composting conditions (Jiang et al., 2015; López-González et al., 2015; Kumari et al., 2015). In this study, composting did not change bacterial richness and frequent or dominant species (Hill number at *q* = 0, 1, and 2), but altered the bacterial community structure. Wan et al. (2021) also reported that composting leads to large changes in the bacterial community structure and the dominant species. They reported that Pseudomonadota (Proteobacteria) and Chloroflexota dominated in cattle manure composts. In this study, Pseudomonadota was also the most abundant bacterial phyla in the composted cow manures, with Bacteroidota or Actinomycetota as the second most abundant phylum. In only one cow manure did the most abundant bacterial species at the onset of the experiment, i.e. *Luteimonas sp.* MC1750, also remain the most abundant after 74 days of composting, although its relative abundance was 3.2 times lower. In the other two composted cow manures, members of *Alkaliflexus* became the dominant genus, with the uncultured *Alkaliflexus* sp. Ai-910 is the dominant species. Min et al. (2022) also found that *Alkaliflexus* was the dominant bacterial genus in dairy cattle manure (20.4%). Until now, only one species of this genus has been isolated, i.e., *Alkaliflexus imshenetskii*, an anaerobic saccharolytic bacteria growing in a pH range of 7.5–10.2, with an optimum around pH 8.5 while oxygen, which was not used as an electron acceptor, reduced growth (Zhilina et al., 2004). The isolates, as described by Zhilina et al. (2004) degraded cellobiose, xylose, maltose, xylan, starch, and pectin but did not degrade cellulose, indicating that they utilized soluble products. The high pH in the cow manure, the capacity of members of *Alkaliflexus* to degrade a wide range of organic molecules, and the anaerobic microsites during composting might have favored them.

Based on 16S ribosomal RNA, Partanen et al. (2010) found a large difference in the bacterial community between a pilot and a full-scale facility 70 km apart when considering the OTUs and species, although they were fed with similarly separated municipal biowaste mixed with wood chips, but less so when considering bacterial genera and phyla. They detected five phyla Actinomycetota, Bacteroidota, Pseudomonadota, and Deinococcota formerly, Deinococcus-Thermus and Bacillota with the latter the most dominant. Except for Deinococcota, these phyla were also the most abundant in this study. Li J. et al. (2020) and Li Z. et al. (2020) investigated the changes in the bacterial community structure during composting of cow manure and sawdust and reported that Pseudomonadota remained dominant after 42 days, the relative abundance of Bacteroidetes increased, while that of Bacillota decreased. In this study, a similar pattern was found after 74 days of composting, Pseudomonadota remained dominant, the relative abundance of Bacteroidetes increased, while that of Bacillota sharply decreased. Li J. et al. (2020) and Li Z. et al. (2020) also found that on day 6 of a pilot-scale composting process of dairy manure, the dominant bacterial genera were *Pseudomonas*, *Sphingobacterium*, and *Bacillus*, while on day 21 *Flavobacterium*, *Myroides*, *Cellvibrio*, *Sphingobacterium*, *Rhodothermus*, *Bacillus*, and *Clostridium* dominated and the relative abundance or *Cellvibrio*. spp. increased to 12.4% at day 42. In this study, only members of *Pseudomonas* were among the most abundant (2.4%) in the cow manure, while at the end of the composting process, none of the bacterial genera reported by Li J. et al. (2020) and Li Z. et al. (2020) were among the most abundant bacterial genera and the relative abundance of Cellvibrio increased from 0.14% to only 0.19%.

Ge et al. (2022) showed that the changes in the bacterial community during composting of cattle manure were affected significantly by total carbon, pH, and initial WC, while Zhu et al. (2019) reported that WC, pH, and C/N ratio were characteristics affecting organic-N transformation by microorganisms during chicken manure, garden waste, and municipal solid waste composting. In this study, the bacterial community was significantly and positively correlated with WC, organic material, and mineral N but not with pH. The pH in the cow manure showed little variation after composting, that is, it changed from 8.6 to 8.4, so its possible effect on the bacterial community was minimal.

#### 4.2.2 Bacterial nitrifiers

Five different AOB genera were detected in the cow manure and the composted cow manure, that is, *Nitrosomonas*, *Nitrosospira*, *Nitrosococcus*, and *Cd. Nitroacidococcus* and *Cd. Nitrosoglobus*, with *Nitrosomonas europaea* (16.03 × 10^−3^%) the most abundant species. The NOB contained three genera, that is, *Cd. Nitrotoga*, *Nitrospira*, and *Nitrobacter*, with members of the latter *N. winogradskyi* (32.50 × 10^−3^%) and *N. hamburgensis* (11.59 × 10^−3^%), the most dominant species. The relative abundance of *Cd. Nitrotoga arctica* (2.30 × 10^−3^%), a cold-adapted NOB (Lücker et al., 2015), was similar to that of *Nitrospira* (*Neomysis japonica*, 2.70 × 10^−3^% and *N. moscoviensis* 5.13 × 10^−3^%). Lücker et al. (2015) also found that the relative abundance of *Cd. Nitrotoga* was similar to that of *Cd. Nitrospira* in different wastewater treatment plants indicates it is a functionally important NOB. Some members of *Cd. N. inopinata* (3.21 × 10^−3^%), complete ammonia oxidizer (comammox) bacteria, were also detected in the cow manure and composted cow manure confirming that their distribution is more widespread than previously thought (Zhang H. et al., 2022; Zhang Y. et al., 2022). Another interesting bacterium involved in the N cycle detected in the cow manure and the composted manure was *A. indicus*—found to be capable of heterotrophic nitrification-aerobic denitrification (Ke et al., 2022). It would be interesting to study the functional activity of these bacteria during composting to determine their contribution to N cycling. Wang et al. (2017) studied anaerobic ammonium-oxidizing (anammox) bacteria by targeting the 16S rRNA gene and the hydrazine oxidase gene (*hzo*) in samples isolated from compost produced from cow manure and rice straw and detected *Cd. Brocadia*, *Cd. Kuenenia*, and *Cd. Scalindua*. In this study, only *Cd. Kuenenia stuttgartiensis* (1.14 × 10^−3^%) was detected, as well as hydrazine synthase subunit beta and gamma, with both showing a 4-fold increase after composting.

Yamamoto et al. (2010) found that the AOB community structure changed during composting, as found in this study. Yan et al. (2015) found that members of *Nitrosomonas* were dominant throughout the entire 28-day composting process of municipal sludge at a field-scaled facility. Sun et al. (2019) composted cattle manure in an aerated vessel and found AOB closely related to *Nitrosomonas* spp., *Nitrosomonas eutropha*, and *Nitrosospira* spp. and uncultured bacteria, with *Nitrosomonas* spp. predominant. In this study, the AOB were also dominated by members of *Nitrosomonas*, and composting for 74 days increased their relative abundance 2.7 times.

The relative abundance of most AOB and NOB decreased after composting which would suggest a decrease in available NH_4_^+^ or NO_2_^−^. Sun et al. (2019) found that WC, total nitrogen (TN), and ammonium concentration were important for the AOB community structure. In this study, NH_4_^+^ and WC content were positively correlated with AOB. The C/N ratio of the organic material in the cow manure was low, so the total N was not a defining factor during composting.

As mentioned before, denitrification occurred during composting as not all NH_4_^+^ detected at the onset of the experiment was converted to NO_3_^−^ after 74 days. The relative abundance of all the genes involved in the reduction of NO_2_^−^ to N_2_, that is, *nirK*, *nirS*, *norB*, *norC*, and *nosZ* showed a small but consistent increase in relative abundance. This would suggest that anaerobic conditions existed during composting, but other factors have also been found to affect the relative abundance of these genes. Meng et al. (2019) reported that the diversity of the *nirK* and *nirS* genes was significantly correlated with NH_4_^+^, that of the *nosZ* gene with pH, and the abundance of the *nirK nirS* and *nosZ* genes with temperature (*p* < 0.05). In this study, the genes involved in the N cycle were correlated positively and significantly with salt content, organic material, and NH_4_^+^ and NO_2_^−^ concentrations. It must be remembered that some NH_3_ might have been lost through volatilization as the cow manure was alkaline which would have affected the correlation between the genes involved in the N cycle and the mineral N content.

#### 4.2.3 Methanotrophs and methylotrophs

The aerobic methanotrophs detected in the cow manure and the composted cow manure belonged to four bacterial groups, that is, α-, β-, and γ-proteobacteria and Verrucomicrobiota. Some of them are obligate methanotrophs, for example, *M. alcaliphilum* (Rozova et al., 2021), but most are facultative methanotrophs and methylotrophs (Farhan Ul Haque et al., 2020). Bacteria capable of anaerobic oxidation of methane coupled with denitrification, such as *Cd. Methylomirabilis oxyfera* members of the phylum Nitrospirota (Cui et al., 2015; Welte et al., 2016), were not detected in the cow manure and the composted cow manure. Additionally, no members of *Cd. Methanoperedens nitroreducens* (Methanosarcinales), archaea capable of anaerobic methane oxidation coupled to nitrate reduction, were found (McIlroy et al., 2023).

Composting of animal manures generally increases the relative abundances of aerobic methanotrophs and methylotrophs as better aeration favors these bacteria. In this study, the relative abundances of all detected methanotrophs and nearly all methylotrophs increased. Also, the relative abundance of the *pmoA* (15.0 times), *pmoB* (13.4 times), and *pmoC* (55.8 times) genes increased substantially after 74 days of composting, which have been reported before (e.g., Sonoki et al., 2013), but possible large variation in *pmoA* values over time, as reported by Sharma et al. (2011) should be considered.

### 4.3 Archaea

#### 4.3.1 The archaeal community structure

Although the archaeal species in the cow manure in this study belonged to nine different phyla, 97.55% belonged to the Euryarchaeota, mostly methanogen species. Ngetich et al. (2022) analyzed six rumen fluid and 42 dung samples from Kenyan and Tanzanian farms using shotgun metagenomics and found that the class Methanomicrobia was the most abundant in the rumen samples (~39%) and dung (~44%) while *M. labreanum* the most abundant (~17%) methanogen species. In this study, the relative abundance of Methanomicrobia in the cow manure was similar (39.15%) to that reported by Ngetich et al. (2022), but higher in the composted cow manure (61.87%). This would suggest that the changes in cow manure characteristics during composting favored the Methanomicrobia. Interestingly, Zhang et al. (2018) studied the effect of dietary forage:concentrate ratios on the archaeal community and found that the relative abundance of *Methanocorpusculum* varied between 32.90 and 61.99% depending on forage:concentrate ratio. As such, feed might partly explain the large variations detected in the relative abundance of *M. labreanum* in the cow manure used in this study. Zhang et al. (2018) reported a high relative abundance of *Methanobrevibacter* (ranging between 26.90 and 49.57%). In this study, the relative abundance of *Methanobrevibacter*, the second most abundant archaeal genus, was 18.10% in the cow manure but dropped to 2.03% after 74 days of composting. Daquiado et al. (2014) reported that *Methanobrevibacter ruminantium* was the dominant archaeal species in rectal dung (63.6%), and barn floor manure (62.4%) of Korean Hanwoo cattle. In this study, the relative abundance of genus *M. ruminantium* was only 2.14%. Consequently, the cow manure characteristics and how the archaeal group responds to the composting process will determine a possible effect on its abundance.

#### 4.3.2 Ammonium oxidizing archaea

Most of the AOA detected in the cow manure and composted cow manure were unknown species or not yet classified, that is, *Cd. Nitrosocaldus*, *Cd. Nitrosopelagicus*, *Cd. Nitrosomarinus*, *Cd. Nitrosotalea*, *Cd. Nitrosotenuis*. Some classified species, such as *Nitrososphaera. viennensis*, *Nitrosopumilus maritimus*, and *Nitrosopumilus piranensis*, were detected in the cow manure and composted cow manure. *N. viennensis* was isolated from soil and grows on ammonia or urea as an energy source and can use higher ammonia concentrations than the marine isolate *N. maritimus* (Tourna et al., 2011). Although *N. viennensis* can grow chemolithoautotrophically, its growth rates increased substantially upon the addition of low amounts of pyruvate or when grown in coculture with bacteria (Tourna et al., 2011). *Nitrosopumilus adriaticus* and *N. piranensis* were isolated from the Adriatic Sea (Bayer et al., 2019). They are mesophilic and neutrophilic that gain energy by oxidizing NH_3_ to NO_2_^−^, and use bicarbonate as a carbon source, while *N. piranensis* can also use urea as a source of ammonia for energy production and growth. The large amounts of NH_4_^+^ in the cow manure and the capacity of AOA to survive in environments with high salt contents explain their large diversity in the cow manure and the composted cow manure. Overall, the relative abundance of AOA increased 1.4-fold, but not all AOA groups were enriched. For instance, the relative abundance of the five *Nitrosopumilus* species decreased after composting, so another factor, for example, the high amount of NH_3_ (Tourna et al., 2011), determined their relative abundance or other AOA or AOB inhibited their growth.

Sun et al. (2019) composted cattle manure in an aerated vessel with and detected five AOA sequences belonging to *Cd. Nitrososphaera gargensis* and to an uncultured archaeon. Yan et al. (2015) found that members of *Cd. N. gargensis* was dominant throughout the entire 28-day composting process of municipal sludge at a field-scaled facility. In this study, *Cd. N. gargensis* was also detected, and its relative abundance decreased after composting for 74 days.

Zou et al. (2023) reported that AOA in estuaries (*Nitrosomarinus*, *Nitrosopumilus*, *Aestuariumsis*, *Nitrosarchaeum*, and *Nitrosopelagicus*-like groups) were affected by salinity, pH, and dissolved oxygen. Sun et al. (2019) reported that high correlations were observed between ammonia, nitrate, and total N and the AOA community. In this study, NH_4_^+^ and WC content were positively correlated with AOA. As mentioned before, the C/N ratio of the organic material in the cow manure was low, so N was not a limiting factor and will have favored the AOA that gains energy from oxidizing NH_3_. Additionally, changes in pH in the cow manure after composting were small, so its possible effect on the AOA must have been small.

#### 4.3.3 Methanogens

Ngetich et al. (2022) studied rumen and dung samples of dairy cows from Kenyan and Tanzanian farms using a shotgun metagenomic approach and found that the class Methanomicrobia was the most abundant in the rumen samples (~39%) and dung (~44%) with *M. labreanum* the most abundant (~17%) methanogen species. Li J. et al. (2020) and Li Z. et al. (2020) investigated the rumen methanogens of three ruminant families, Cervidae (deer), Bovidae (bovid), and Moschidae (musk deer), and found that members of *Methanobrevibacter* spp. were the most widespread methanogens. Malik et al. (2024) reported that Euryarchaeota dominated the archaeal community in cattle and buffaloes, with Methanobacteriales the dominant order and *Methanobrevibacter* the most prevalent genus of methanogens, but members of Methanosarcinales, Methanococcales, Methanomicrobiales, and Methanomassiliicoccales were also detected. In this study, Euryarchaeota also dominated in the cow manure and composted cow manure with the methanogens *Methanocorpusculum*, *Methanosarcina*, and *Methanobrevibacter*, the prevalent archaeal genera, although the genus that dominated varied with the location where the cow manure was collected. Differences in diet are known to alter the archaeal community structure and the dominant methanogen in cow manure (Vaidya et al., 2020).

Guo et al. (2021) studied the methanogens in fresh pig manure and wheat straw composted for 42 days and found that the dominant methanogenic genera were members of *Methanobrevibacter*, *Methanobacterium*, *Methanothermobacter*, and *Methanocorpusculum*, which accounted for 74.25–99.46% of the total sequences. They found that the relative abundance of *Methanocorpusculum* and *Methanobrevibacter* decreased after 42 days of composting, but the latter remained the dominant methanogenic genus. In this study, the relative abundance of *Methanocorpusculum*, however, increased 34.1 times (mean of the three different cow manures) and that of *Methanobrevibacter* 1.5 times. This confirms that the cow manure characteristics and composting conditions might determine which microbial groups might be enriched.

Yu et al. (2023) studying the effects of inoculation with lignocellulose-degrading microorganisms on the degradation of organic matter during composting, found that organic material and temperature were the main factors that affected the bacterial and methanogen community structures. In this study, the relative abundance of methanogens was significantly and positively correlated with salt content, that is, EC, WC, and WHC emitted CO_2_ and NO_3_^−^ content. As anaerobes, the relative abundance of methanogens will be defined by anaerobic conditions, which, as mentioned before, is related to O_2_ availability controlled by WC and microbial activity.

The relative abundance of methanogens and the methylcoenzyme M reductase (*mcrA*) gene increased when the cow manure was composted, but the effect on the different methanogenic groups was different. This indicated that anaerobic conditions existed during composting, but changes in organic material composition and environmental conditions, for example, temperature, affected methanogenic groups differently (Yu et al., 2023). Chen et al. (2014) studied methanogens in manure and found that after a 50-day period, the relative abundance of *Methanosarcina* decreased and that of *Methanobrevibacter* increased, but manure management, that is, windrow composting or solid storage affected the methanogenic community. In this study, we found an opposite result, that is, the relative abundance of Methanosarcina increased 2.1 times (mean of the three different cow manures and composted cow manures), and that of *Methanobrevibacter* decreased 1.6 times, confirming that the cow manure characteristics and composting conditions determine which microbial groups will be enriched. For instance, Rademacher et al. (2012) reported that when the temperature in a leach bed reactor was 55°C, members of the Methanobacteriales dominated in the downstream anaerobic filter reactor, whereas at higher temperatures in the leach bed reactor (75°C) Methanosarcinales prevailed. As such, the origin of the compost, for example its composition, but also the characteristics of the methanogenic group will determine a possible effect of the composting process. For instance, the relative abundance of *Methanosarcina* increased in two of the composted cow manures, that is, 1.5 and 6.5 times, but decreased 11.3 times in the other one.

### 4.4 Comparison between the metabolic functions and the archaeal and bacterial communities

In earlier studies based on the 16S rRNA gene, we found that the variations in putative metabolic functions between samples of the same treatment and also between the different treatments were much smaller than those detected between bacterial groups (Zarco-González et al., 2023). However, these metabolic functions were putative and based on taxonomic classification. As such, the question remained if this was a “real” phenomenon or an artifact. Many factors, such as microbial interactions and resource availability, can affect the expression of genes related to a certain functional activity. This means that while different archaeal and bacterial groups can perform the same metabolic function, they may not all be active in the same environment at the same time and may even compete sometimes. It would be interesting to determine changes in mRNA content so that not only genes in microorganisms but also their transcription can be studied.

Xia et al. (2022) studied the assembly of functional genes and taxa in soil and the gut of the earthworm under vanadium stress and found that the taxa were more sensitive to stress in soil compared with functional genes but not in the earthworm gut. This suggested the existence of bacterial functional redundancy in soil but not in the earthworm gut. Miralles et al. (2021) studied the functional and taxonomic effects of organic amendments on the restoration of semiarid quarry soil. They reported that both taxonomic and functional metagenomic sequencing profiling clearly separated the organically amended soil from the unamended control samples. They also stated that although the taxonomic differences were clear, the functional redundancy was higher than expected.

The study reported here based on shotgun metagenomics confirmed that the variation in the relative abundance of bacterial and archaeal groups was much larger than that of genes within replicated samples of the same cow manure and compost, but also between the cow manures and the composted cow manures. This confirms that changes in archaeal and bacterial groups were more stochastic than those of the metabolic functions, that is, different archaeal and bacterial groups can perform the same metabolic function, and, as such, there was a clear functional redundancy (Louca et al., 2018). This might be important in composting, where maintaining a stable functional capacity for processes such as nitrogen and carbon cycling is crucial. As such, although the bacterial and archaeal community shows large variations, important metabolic functions were less variable.

## 5 Conclusion

In this study, we collected cow manure from three local farmers with significant differences in water, NH_4_^+^ and NO_2_^−^ content, and WHC. We assumed that the archaeal and bacterial community structures would be different between them but that composting would largely reduce these differences. The archaeal and bacterial communities were indeed different in the three cow manures at the onset of the experiment, and these might be due to cow characteristics, type of feed, treatments applied to the cattle, and storage conditions of the cow manure. Composting affected different bacterial and archaeal groups strongly, and the microbial community structures remained different even after 74 days. As such, bacterial and archaeal communities, but also the composition of specific groups, such as AOB and archaea, NOB, methanogens, methanotrophs, and methylotrophs, changed during composting, but the changes were defined by the origin of the cow manure and persisted independent of composting. A similar pattern emerged when considering bacterial genes, even though the variations in their relative abundance were much smaller. This means that the possible use of plant growth-promoting microorganisms in composted cow manure to improve crop yields will depend heavily on the type and origin of the animal waste. As such, local farmers who might want to use their composted cow manure might have a product that is highly diverse depending on the cow’s diet and cow manure storage. Advising on how to use the composted cow manure might depend not only on soil characteristics and the crop cultivated but also on the obtained compost.

It must be remembered, however, that the composting of the cow manure in this study occurred under controlled conditions in columns in the greenhouse, so it would be interesting to investigate if composting of animal wastes under different conditions, that is, in heaps where higher temperatures can be reached, under dryer conditions with regular mixing and better aeration and for more than 74 days, would have the same effect on the microbial communities and their metabolic functions.

## Data Availability

The datasets presented in this study can be found in online repositories. The names of the repository/repositories and accession number(s) can be found in the article/Supplementary material.

## Footnotes

^1^ https://sourceforge.net/projects/bbmap/

^2^ https://github.com/tseemann/prokka

^3^ https://github.com/qichao1984/NCyc

## Supplementary material

SUPPLEMENTARY FIGURE S1 Pictures of the cow manures, composting of the cow manures, characterization and DNA extraction, and a map of Tlaxcala (Mexico) with the locations of the sampling spots.

SUPPLEMENTARY FIGURE S2 An example of an 0.8% agarose gel indicating the integrity of the extracted DNA.

SUPPLEMENTARY FIGURE S3 Box plot with Hill numbers at *q* = 0, 1 and 2 of **(A)** bacterial and **(B)** archaeal species and **(C)** genes in the cow manures at the beginning (Start) and after 74 days composting (End).

SUPPLEMENTARY FIGURE S4 Barplots with the relative abundance (%) of the **(A)** bacterial and **(B)** archaeal phyla, and **(C)** bacterial and **(D)** archaeal genera in the cow manures from three location at the start of the experiment (St) and after 74 days composting (En).

SUPPLEMENTARY FIGURE S5 Volcano plot comparing the relative abundance of **(A)** bacterial and **(B)** archaeal species and **(C)** genes in the cow manure at the onset of the experiment versus the cow manures composted for 74 days. The expected *p*-value of the Kruskal–Wallis test is given in the *y*-axis and the effect size is given in the *x*-axis (Gloor et al., 2017). The effect size, which is defined as the difference between groups divided by the maximum dispersion within group A or B, was calculated with the ALDEx2 package using the aldex.ttest argument. A negative value indicates that the relative abundance of the microbial group was higher in the cow manures than in the cow manures composted for 74 days and a positive value the opposite. Vertical lines indicate large effects size (≤ −0.8, ≥0.8) and very large effect sizes (≤ −1.3, ≥1.3) (Kim, 2015).

SUPPLEMENTARY FIGURE S6 Variance in the relative abundance of **(A)** bacterial and **(B)** archaeal species and **(C)** genes (%) in the three samples of cow manure (*n* = 3) from three different locations at the start of the experiment (Start) and after 74 days composting (End).

SUPPLEMENTARY FIGURE S7 Variance in the relative abundance of bacterial and archaeal species and genes (%) in the cow manures from three different locations at the start of the experiment (Start) and after 74 days composting (End).

SUPPLEMENTARY FIGURE S8 Ratio between the relative abundance of the 20 most abundant **(A)** bacterial and **(B)** archaeal species and **(C)** genes in the cow manure collected at three locations and the average of these three locations (all) at the onset of the experiment versus that in the cow manure after 74 days composting. Ratio >2 times larger (), >2 times larger or >2 times smaller () and >2 times smaller (). First, when the relative abundance was larger at the onset than after 74 days composting, the ratio was calculated as—(relative abundance at the onset—relative abundance after 74 days composting)/(relative abundance after 74 days composting). Second, when the relative abundance was larger after 74 days composting than at the onset, the ratio was calculated as (relative abundance after 74 days composting − relative abundance at the onset)/(relative abundance at the onset).
