# Supplementary figures and images for: The archaeal and bacterial community structure in composted cow manures is defined by the original populations: a shotgun metagenomic approach
Source: Front Microbiol. 2024 Nov 1;15:1425548. doi: 10.3389/fmicb.2024.1425548 (PMC11583985; doi:10.3389/fmicb.2024.1425548)

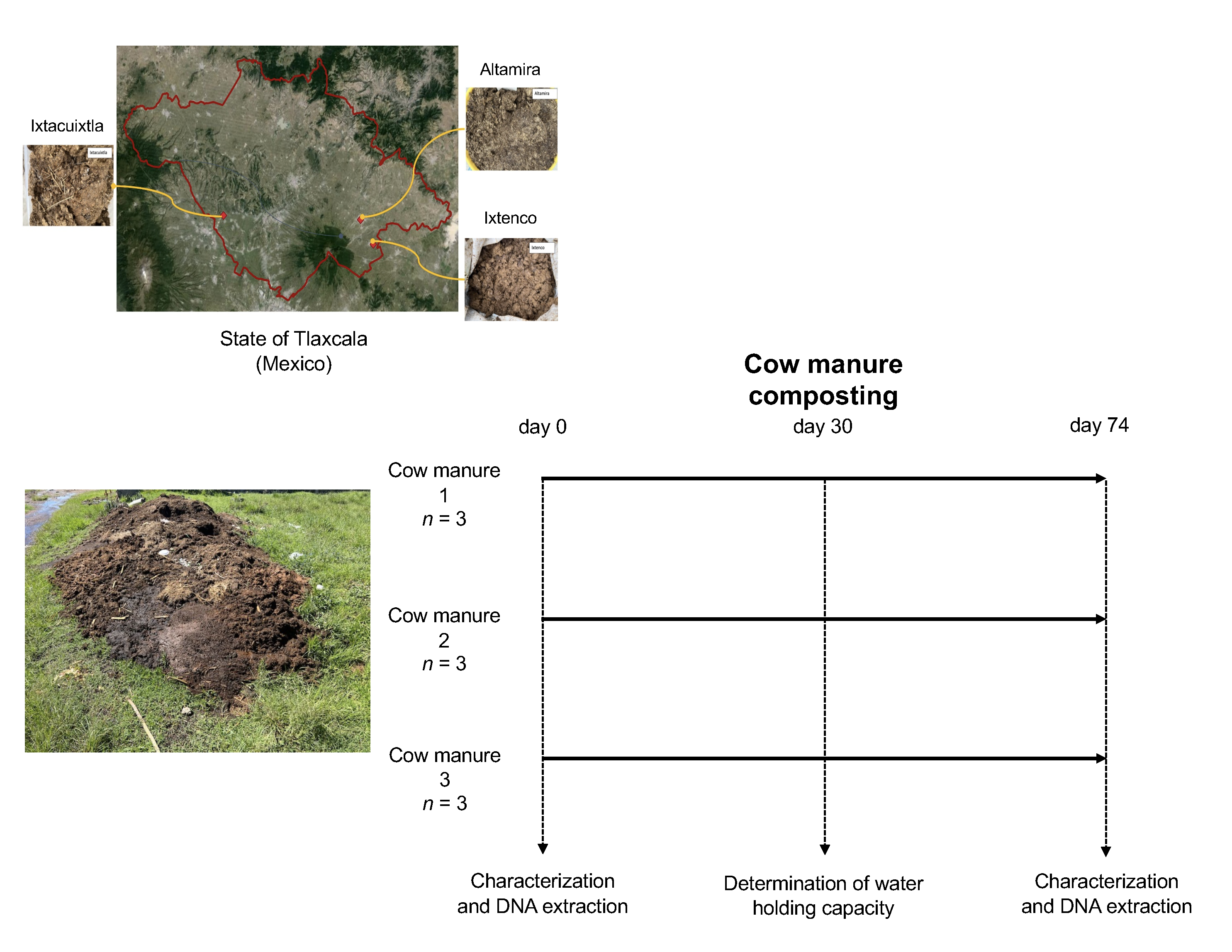

Supplement: SUPPLEMENTARY FIGURE S1 — Pictures of the cow manures, composting of the cow manures, characterization and DNA extraction, and a map of Tlaxcala (Mexico) with the locations of the sampling spots. [file Image_1.TIFF]

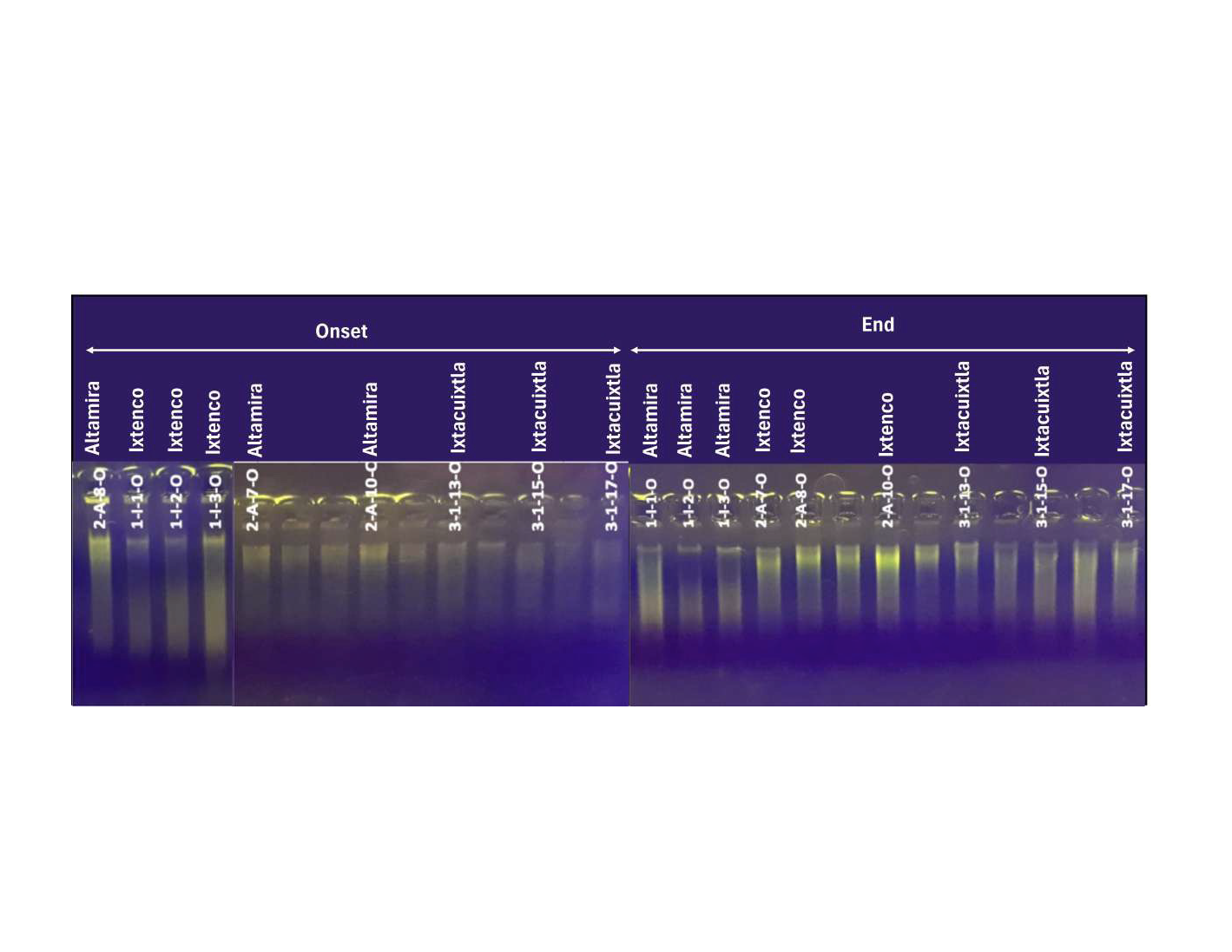

Supplement: SUPPLEMENTARY FIGURE S2 — An example of an 0.8% agarose gel indicating the integrity of the extracted DNA. [file Image_2.TIFF]

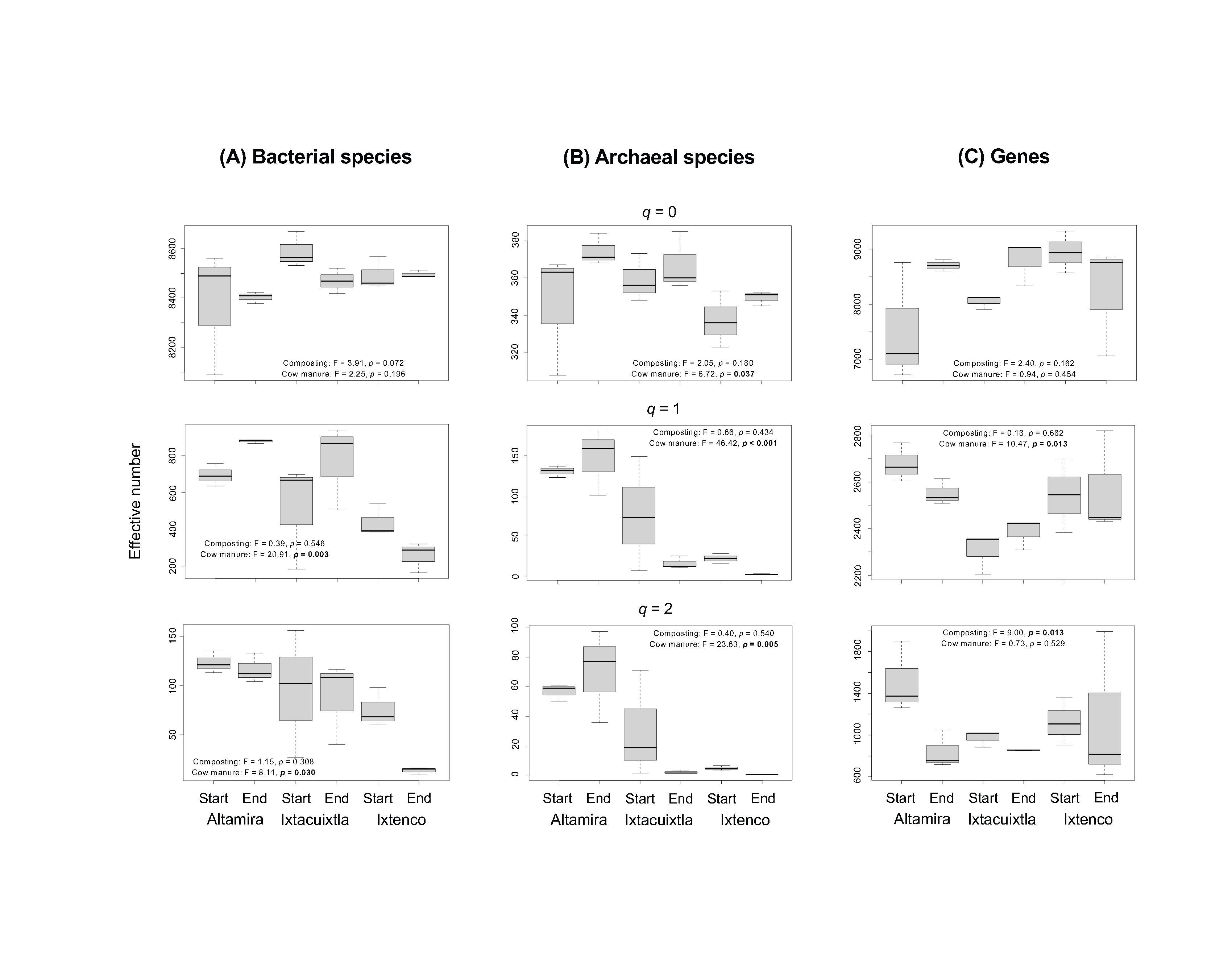

Supplement: SUPPLEMENTARY FIGURE S3 — Box plot with Hill numbers at q = 0, 1 and 2 of (A) bacterial and (B) archaeal species and (C) genes in the cow manures at the beginning (Start) and after 74 days composting (End). [file Image_3.TIFF]

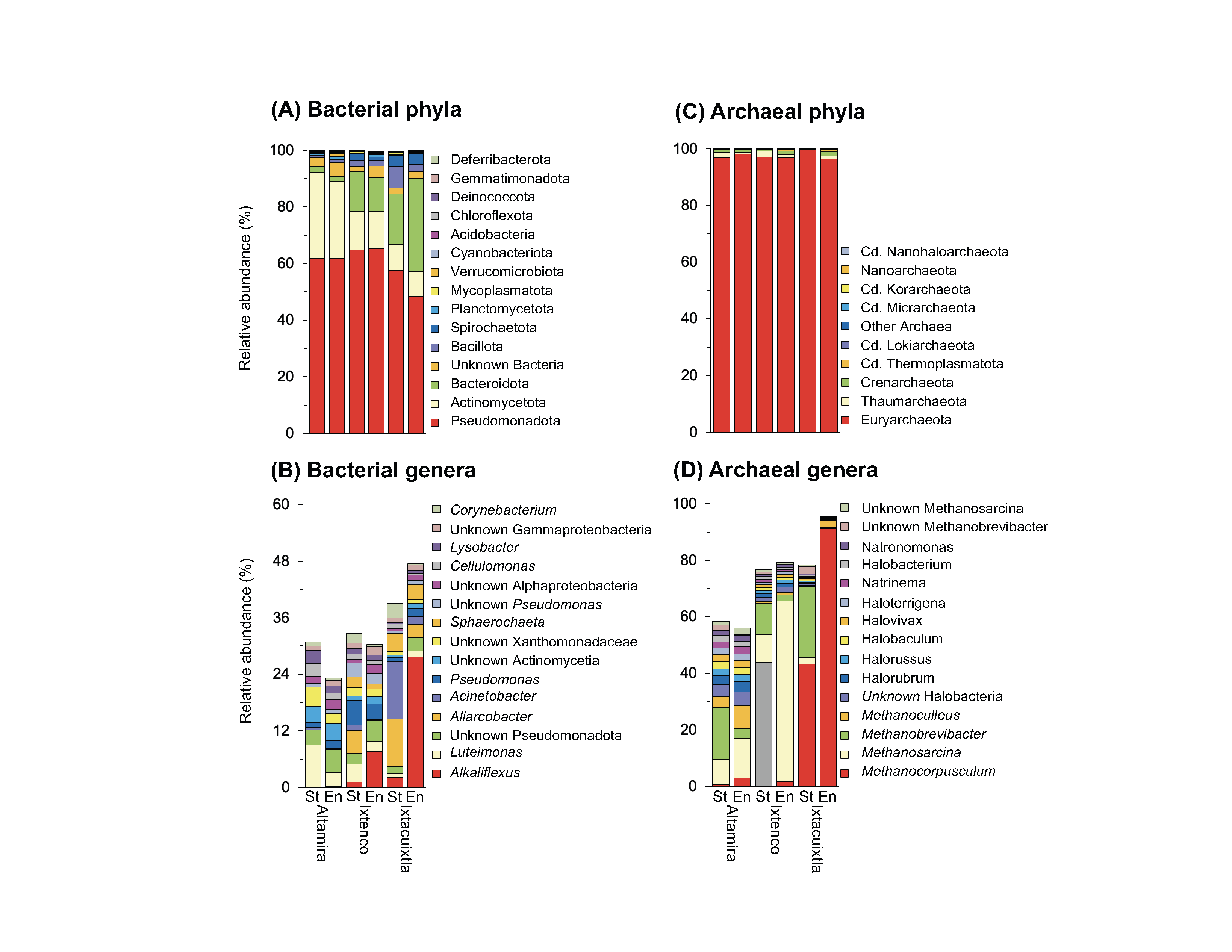

Supplement: SUPPLEMENTARY FIGURE S4 — Barplots with the relative abundance (%) of the (A) bacterial and (B) archaeal phyla, and (C) bacterial and (D) archaeal genera in the cow manures from three location at the start of the experiment (St) and after 74 days composting (En). [file Image_4.TIFF]

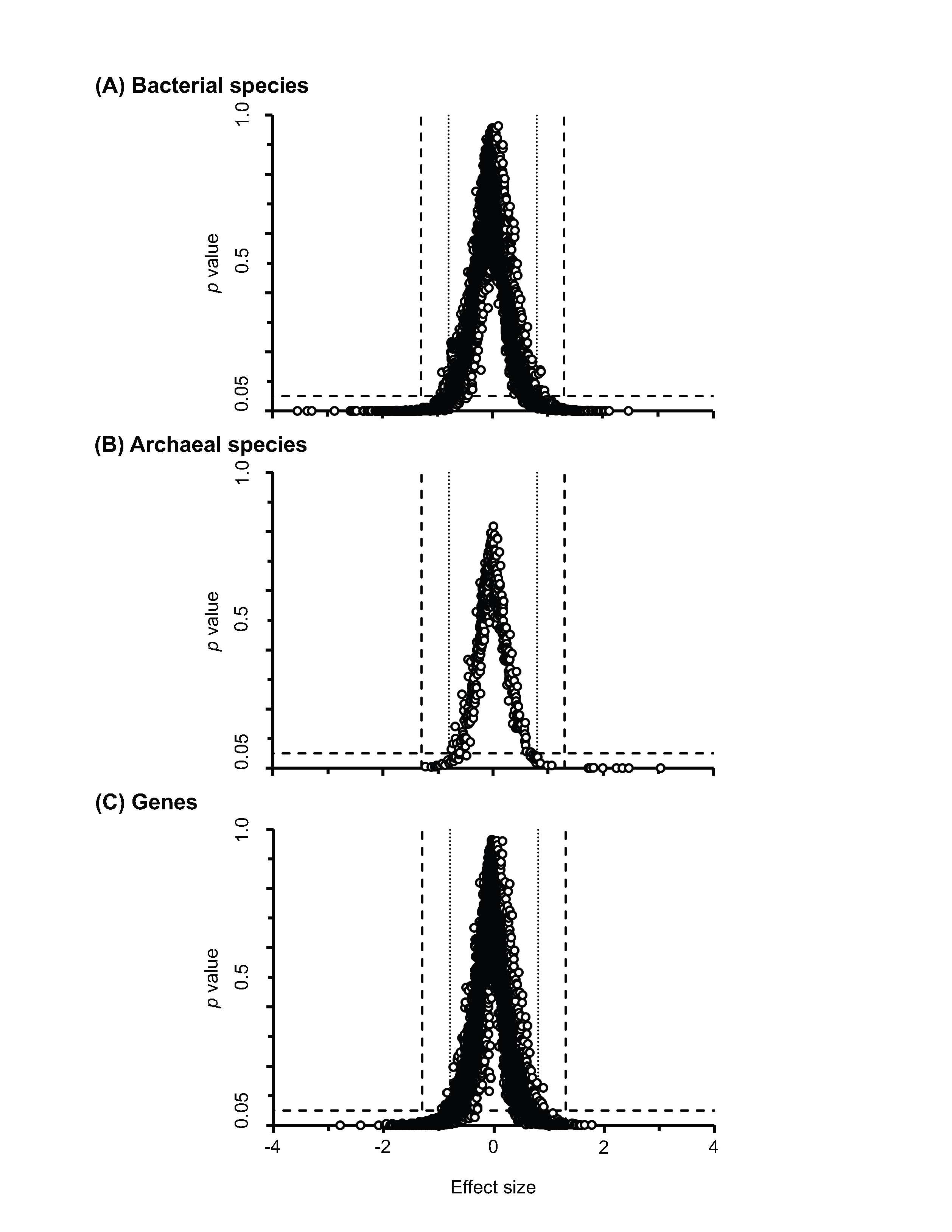

Supplement: SUPPLEMENTARY FIGURE S5 — Volcano plot comparing the relative abundance of (A) bacterial and (B) archaeal species and (C) genes in the cow manure at the onset of the experiment versus the cow manures composted for 74 days. The expected p-value of the Kruskal–Wallis test is given in the y-axis and the effect size is given in the x-axis (Gloor et al., 2017). The effect size, which is defined as the difference between groups divided by the maximum dispersion within group A or B, was calculated with the ALDEx2 package using the aldex.ttest argument. A negative value indicates that the relative abundance of the microbial group was higher in the cow manures than in the cow manures composted for 74 days and a positive value the opposite. Vertical lines indicate large effects size (≤ −0.8, ≥0.8) and very large effect sizes (≤ −1.3, ≥1.3) (Kim, 2015). [file Image_5.TIFF]

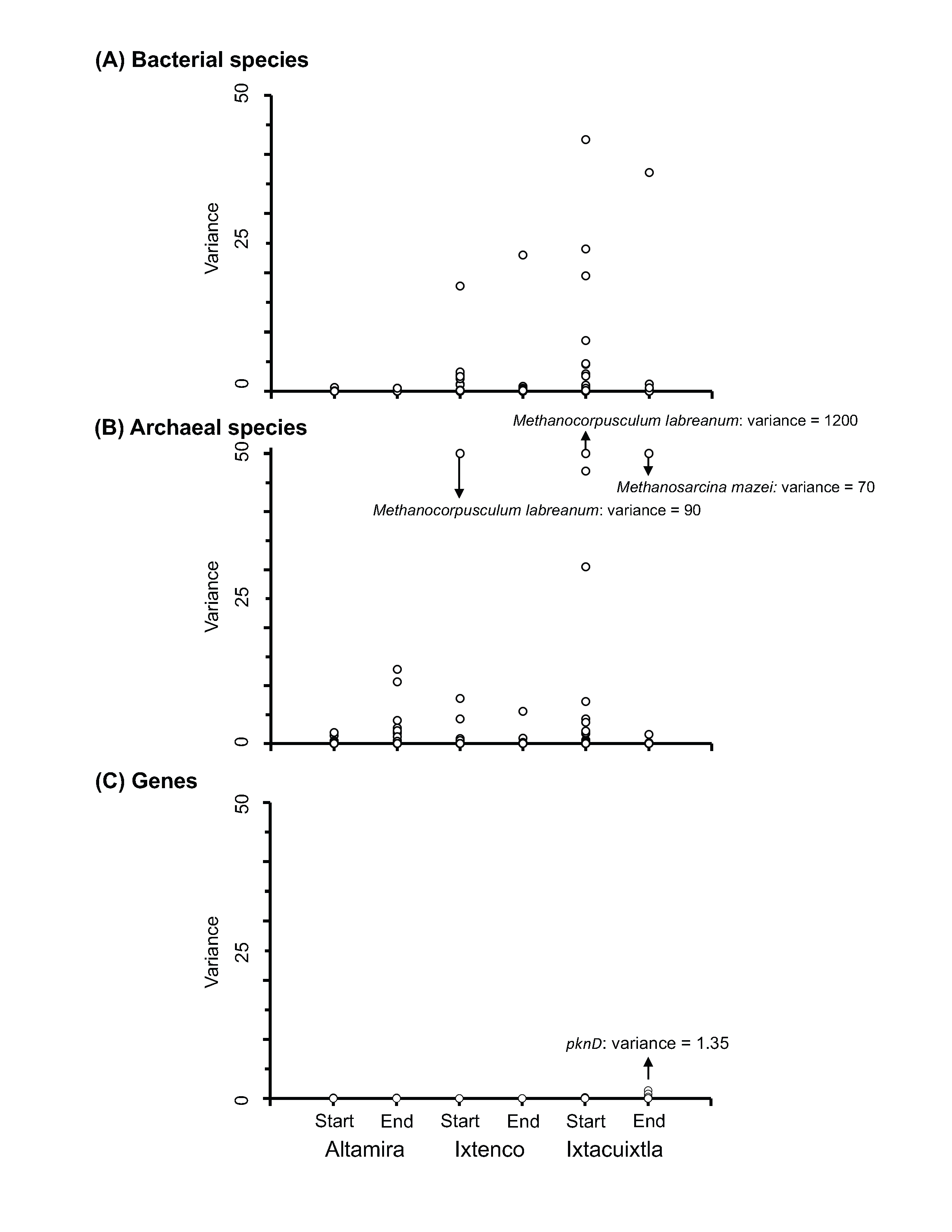

Supplement: SUPPLEMENTARY FIGURE S6 — Variance in the relative abundance of (A) bacterial and (B) archaeal species and (C) genes (%) in the three samples of cow manure (n = 3) from three different locations at the start of the experiment (Start) and after 74 days composting (End). [file Image_6.TIFF]

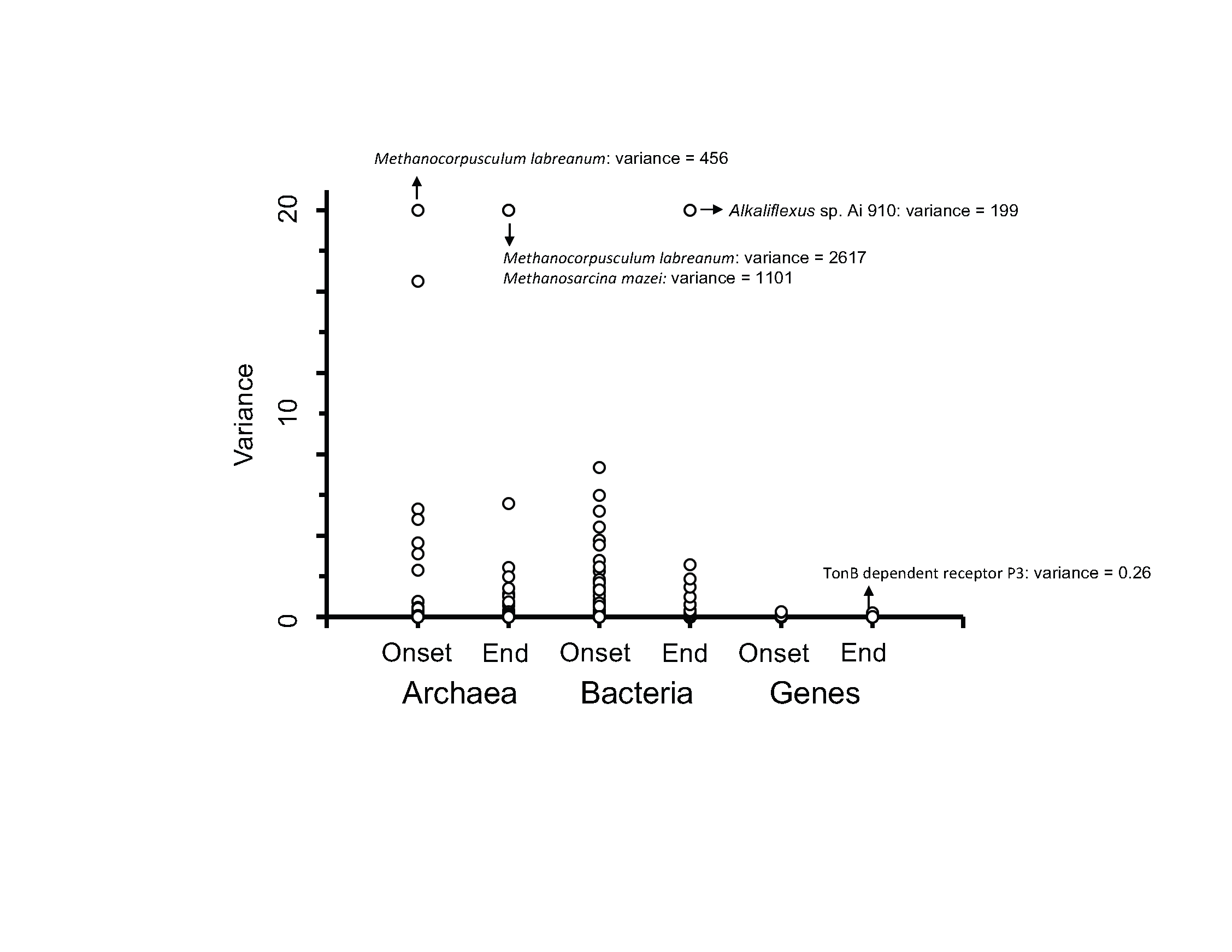

Supplement: SUPPLEMENTARY FIGURE S7 — Variance in the relative abundance of bacterial and archaeal species and genes (%) in the cow manures from three different locations at the start of the experiment (Start) and after 74 days composting (End). [file Image_7.TIFF]

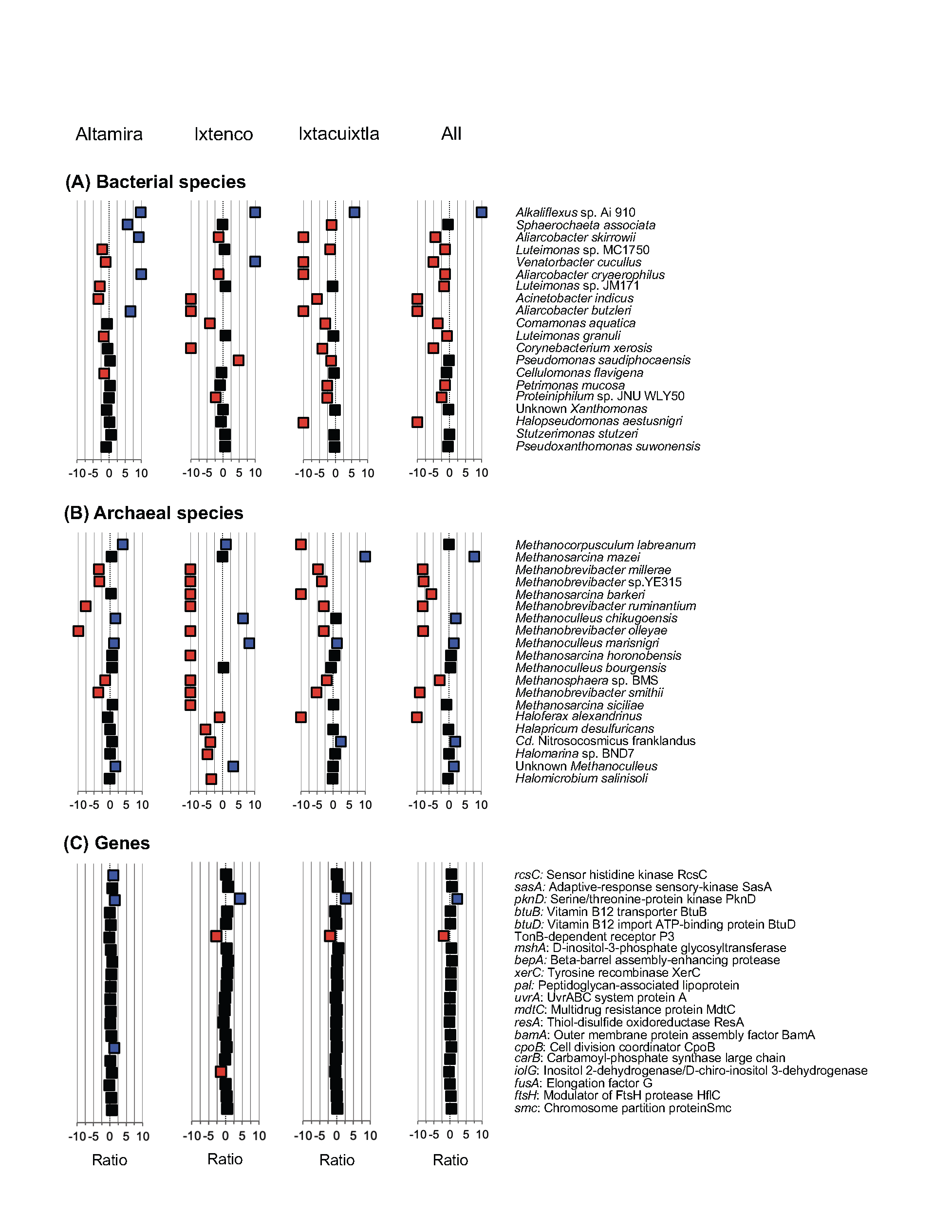

Supplement: SUPPLEMENTARY FIGURE S8 — Ratio between the relative abundance of the 20 most abundant (A) bacterial and (B) archaeal species and (C) genes in the cow manure collected at three locations and the average of these three locations (all) at the onset of the experiment versus that in the cow manure after 74 days composting. Ratio >2 times larger (), >2 times larger or >2 times smaller () and >2 times smaller (). First, when the relative abundance was larger at the onset than after 74 days composting, the ratio was calculated as—(relative abundance at the onset—relative abundance after 74 days composting)/(relative abundance after 74 days composting). Second, when the relative abundance was larger after 74 days composting than at the onset, the ratio was calculated as (relative abundance after 74 days composting − relative abundance at the onset)/(relative abundance at the onset). [file Image_8.TIFF]
