# Supplementary material for: The archaeal and bacterial community structure in composted cow manures is defined by the original populations: a shotgun metagenomic approach
Source: Front Microbiol. 2024 Nov 1;15:1425548. doi: 10.3389/fmicb.2024.1425548 (PMC11583985; doi:10.3389/fmicb.2024.1425548)
Supplement: Supplementary file 9 [file Table_1.DOCX]

**Table S1**. Mantel test between different microbial groups and genes, and characteristics of the cow manures.

| —————————————————————————————————————————————————————————————— | | | | | | | | | | | | | | | | | | | | | | |
| --- | --- | --- | --- | --- | --- | --- | --- | --- | --- | --- | --- | --- | --- | --- | --- | --- | --- | --- | --- | --- | --- | --- |
|  |  | | EC ^a^ | | WHC ^b^ | | WC ^c^ | | Organic C | | Total N | |  | | CO_2_ ^d^ | | Ammonium | | Nitrite | | Nitrate | |
|  | pH | | (dS m^-1^) | | —————— (g kg^-1^ dry soil) ——————— | | | | | | | | C/N ratio | | (%) | | ———— (mg kg^-1^ dry soil) ———— | | | | | |
|  | ———— | | ———— | | ———— | | ———— | | ———— | | ———— | | ———— | | ———— | | ———— | | ———— | | ———— | |
| —————————————————————————————————————————————————————————————— | | | | | | | | | | | | | | | | | | | | | | |
| Group | r ^e^ | *p ^f^* | r | *p* | r | *p* | r | *p* | r | *p* | r | *p* | r | *p* | r | *p* | r | *p* | r | *p* | r | *p* |
| —————————————————————————————————————————————————————————————— | | | | | | | | | | | | | | | | | | | | | | |
| Bacteria | -0.08 | 0.718 | -0.01 | 0.462 | 0.53 | **0.001** ^g^ | 0.54 | **0.001** | -0.25 | **0.021** | -0.01 | 0.440 | 0.48 | **0.001** | 0.22 | **0.018** | 0.54 | **0.002** | 0.52 | **0.001** | -0.21 | 0.980 |
| Methylotrophs | 0.01 | 0.415 | 0.06 | 0.217 | 0.37 | **0.004** | 0.25 | **0.012** | 0.27 | **0.011** | 0.03 | 0.332 | 0.39 | **0.001** | 0.43 | **0.001** | 0.49 | **0.001** | 0.42 | **0.002** | 0.07 | 0.232 |
| Methanotrophs | 0.07 | 0.222 | 0.08 | 0.153 | 0.25 | **0.015** | 0.13 | 0.074 | 0.22 | **0.021** | 0.02 | 0.359 | 0.31 | **0.002** | 0.50 | **0.001** | 0.42 | **0.001** | 0.33 | **0.003** | 0.23 | **0.022** |
| AOB ^h^ | 0.15 | 0.082 | -0.01 | 0.499 | 0.18 | **0.044** | 0.16 | **0.046** | 0.13 | 0.082 | -0.14 | 0.937 | 0.11 | 0.143 | 0.46 | **0.001** | 0.22 | **0.029** | 0.24 | **0.019** | -0.01 | 0.477 |
| NOB ^i^ | -0.09 | 0.720 | 0.12 | 0.105 | 0.03 | 0.357 | 0.14 | 0.074 | -0.14 | 0.952 | -0.01 | 0.450 | -0.03 | 0.563 | 0.15 | 0.065 | -0.14 | 0.863 | -0.06 | 0.635 | 0.16 | 0.159 |
| Archaea | 0.01 | 0.408 | 0.33 | **0.004** | 0.32 | **0.004** | 0.42 | **0.001** | 0.16 | 0.052 | 0.03 | 0.330 | 0.23 | **0.017** | 0.08 | 0.141 | 0.03 | 0.307 | 0.12 | 0.098 | 0.30 | **0.007** |
| Methanogens | 0.13 | 0.106 | 0.33 | **0.003** | 0.24 | **0.016** | 0.31 | **0.005** | 0.16 | 0.055 | 0.05 | 0.285 | 0.12 | 0.113 | 0.17 | **0.043** | -0.06 | 0.726 | -0.01 | 0.509 | 0.39 | **0.001** |
| AOA ^j^ | 0.12 | 0.168 | 0.06 | 0.228 | 0.22 | **0.048** | 0.21 | **0.024** | 0.15 | 0.079 | 0.07 | 0.267 | 0.22 | **0.047** | 0.30 | **0.003** | 0.51 | **0.001** | 0.40 | **0.001** | 0.23 | 0.062 |
| Genes | -0.05 | 0.633 | 0.17 | **0.041** | 0.35 | **0.003** | 0.48 | **0.001** | 0.25 | **0.013** | -0.02 | 0.550 | 0.26 | **0.017** | 0.16 | **0.042** | 0.25 | **0.031** | 0.31 | **0.007** | 0.07 | 0.265 |
| N genes contigs | -0.02 | 0.567 | 0.19 | **0.030** | 0.56 | **0.001** | 0.61 | **0.001** | 0.34 | **0.003** | -0.06 | 0.721 | 0.38 | **0.001** | 0.04 | 0.567 | 0.46 | **0.001** | 0.59 | **0.001** | -0.11 | 0.857 |
| —————————————————————————————————————————————————————————————— | | | | | | | | | | | | | | | | | | | | | | |
| ^a^ EC: Electrolytic conductivity, ^b^ WHC: Water holding capacity, ^c^ WC: Water content, ^d^ CO_2_: Percentage of carbon emitted as carbon dioxide during a 7 day aerobic incubation at 22±2^o^C, ^e^ r: Mantel statistic, ^f^ p: significance, ^g^ values in bold are significant at *p* < 0.05, ^h^ AOB: Ammonium oxidizing bacteria, ^i^ NOB: Nitrite oxidizing archaea, ^j^ AOA: Ammonium oxidizing archaea. | | | | | | | | | | | | | | | | | | | | | | |
| —————————————————————————————————————————————————————————————— | | | | | | | | | | | | | | | | | | | | | | |
