# Supplementary material for: The archaeal and bacterial community structure in composted cow manures is defined by the original populations: a shotgun metagenomic approach
Source: Front Microbiol. 2024 Nov 1;15:1425548. doi: 10.3389/fmicb.2024.1425548 (PMC11583985; doi:10.3389/fmicb.2024.1425548)
Supplement: Supplementary file 10 [file Table_2.DOCX]

**Table S2.** Relative abundance of genes involved in the most important processes involved in nitrogen cycling at the onset of the experiment, i.e. the cow manures, and after 74 days of composting.

| ——————————————————————————————————————————————————————— | | | | | | | |
| --- | --- | --- | --- | --- | --- | --- | --- |
|  | | | | Relative abundance  — (% × 10^-3^) — | |  |  |
| Process, genes and metabolic function | | | | Onset | End | Effect size ^a^ | p value ^b^ |
| ——————————————————————————————————————————————————————— | | | | | | | |
| Nitrification | | | |  |  |  |  |
|  | Ammonium oxidation | | |  |  |  |  |
|  | | *amoA1* | Ammonia monooxygenase alpha subunit | ND ^c^ | 0.190 | 0.5 | 0.210 |
|  | | *amoB1* | Ammonia monooxygenase beta subunit | ND | 0.377 | 1.3 | **0.006** ^d^ |
|  | Hydroxylamine oxidation | | |  |  |  |  |
|  | | *hao1* | Hydroxylamine oxidoreductase | 0.010 | 0.520 | 1.1 | **0.008** |
|  | Nitrite oxidoreductase | | |  |  |  |  |
|  | | *nrxA* | **Nitrite oxidoreductase, alpha subunit** | ND | ND | ND | ND |
|  | | *nrxB* | **Nitrite oxidoreductase, beta subunit** | ND | ND | ND | ND |
| Assimilatory nitrate reduction | | | | | |  |  |
|  | | *nasA* | Nitrate transporter | 11.405 | 20.796 | 0.5 | 0.124 |
|  | | *nasB* | Assimilatory nitrate reductase electron transfer subunit | 0.506 | 1.960 | 0.6 | 0.123 |
|  | | *nasC* | Assimilatory nitrate reductase catalytic subunit | 7.438 | 13.523 | 0.6 | 0.155 |
|  | | *nasR* | Nitrate regulatory protein | 2.070 | 2.713 | 0.3 | 0.286 |
| Dissimilatory nitrate reduction | | | | | |  |  |
|  | | *napA* | Periplasmic nitrate reductase | 37.291 | 57.095 | 0.6 | 0.097 |
|  | | *napB* | Periplasmic nitrate reductase electron transfer subunit | 3.078 | 2.389 | -0.1 | 0.802 |
|  | | *napC* | Cytochrome c-type protein NapC | 6.303 | 3.895 | -0.3 | 0.336 |
|  | | *napD* | Chaperone NapD | 3.954 | 2.246 | -0.5 | 0.276 |
|  | | *napF* | Ferredoxin-type protein NapF | 23.831 | 28.281 | 0.1 | 0.744 |
|  | | *napG* | Ferredoxin-type protein NapG | 2.420 | 1.155 | -0.4 | 0.088 |
|  | | *napH* | Ferredoxin-type protein NapH | 1.991 | 1.499 | 0.0 | 0.867 |
|  | | *narB* | Nitrate reductase | 3.131 | 9.613 | 0.7 | **0.006** |
|  | | *narG* | Nitrate reductase alpha subunit | 21.002 | 23.671 | -0.1 | 0.790 |
|  | | *narH* | Respiratory Nitrate reductase 1 beta chain | 6.279 | 6.021 | 0.0 | 0.881 |
|  | | *narI* | Respiratory Nitrate reductase 1 gamma chain | 3.031 | 2.528 | 0.0 | 0.805 |
|  | | *narJ* | Nitrate reductase molybdenum cofactor assembly chaperone NarJ | 2.499 | 2.772 | 0.1 | 0.644 |
| ——————————————————————————————————————————————————————— | | | | | | | |

**Table S2.** Continued

| ——————————————————————————————————————————————————————— | | | | | | |
| --- | --- | --- | --- | --- | --- | --- |
|  | *narK* | Nitrate/nitrite transporter NarK | 9.012 | 8.135 | -0.2 | 0.491 |
|  | *narK2* | putative Nitrate/nitrite transporter NarK2 | 0.940 | 0.954 | 0.0 | 0.807 |
|  | *narL* | Nitrate/nitrite response regulator protein NarL | 3.985 | 4.950 | 0.0 | 0.852 |
|  | *narQ* | Nitrate/nitrite sensor protein NarQ | 2.390 | 2.467 | 0.0 | 0.831 |
|  | *narT* | putative Nitrate transporter NarT | 3.995 | 8.037 | -0.8 | 0.052 |
|  | *narU* | Nitrate/nitrite transporter NarU | 0.693 | 0.061 | 0.7 | 0.083 |
|  | *narV* | Respiratory Nitrate reductase 2.gamma chain | 0.465 | 0.856 | 0.1 | 0.662 |
|  | *narW* | putative Nitrate reductase molybdenum cofactor assembly chaperone NarW | 0.638 | 0.278 | 0.0 | 0.779 |
|  | *narX* | Nitrate reductase-like protein NarX | 8.793 | 7.413 | 0.1 | 0.933 |
|  | *narY* | Respiratory Nitrate reductase 2 beta chain | 4.485 | 3.833 | -0.2 | 0.685 |
|  | *narZ* | Respiratory Nitrate reductase 2 alpha chain | 2.091 | 1.605 | -0.3 | 0.337 |
| Dissimilatory nitrite reduction | | | | |  |  |
|  | *nrfA* | Cytochrome c nitrite reductase subunit NrfA | 24.918 | 19.431 | -0.5 | 0.176 |
|  | *nrfB* | Cytochrome c-type protein NrfB | 0.074 | ND | -0.2 | 0.436 |
|  | *nrfG* | Formate-dependent Nitrite reductase complex subunit NrfG | 0.112 | 0.137 | -0.2 | 0.530 |
|  | *nrfH* | Cytochrome c-type protein NrfH | 7.030 | 6.352 | -0.5 | 0.257 |
| Assimilatory nitrite reductase | | |  |  |  |  |
|  | *nasD* | Nitrite reductase [NAD(P)H] | 25.167 | 38.575 | 0.7 | **0.042** |
|  | *nasE* | Assimilatory nitrite reductase [NAD(P)H] small subunit | 1.352 | 1.029 | 0.1 | 0.529 |
|  | *nirC* | Nitrite transporter NirC | 1.801 | 2.565 | -0.2 | 0.520 |
|  | *nirD* | Nitrite reductase (NADH) small subunit | 2.671 | 1.942 | 0.0 | 0.856 |
| Nitrate transport | | |  |  |  |  |
|  | *nrtA* | Nitrate/nitrite binding protein NrtA | 8.261 | 12.535 | 0.4 | 0.171 |
|  | *nrtB* | Nitrate import permease protein NrtB | 2.986 | 4.399 | 0.7 | 0.154 |
|  | *nrtC* | Nitrate import ATP-binding protein NrtC | 1.723 | 2.005 | 0.4 | 0.190 |
|  | *nrtD* | Nitrate import ATP-binding protein NrtD | 10.150 | 13.086 | 0.3 | 0.369 |
|  | *nrtP* | Nitrate/nitrite transporter NrtP | 4.876 | 7.954 | 0.3 | 0.274 |
| Nitrogen fixation | | |  |  |  |  |
|  | *nifA* | Nif-specific regulatory protein | 2.117 | 4.841 | 0.4 | **0.020** |
|  | *nifB* | FeMo.cofactor biosynthesis protein NifB | 1.511 | 6.567 | 0.5 | 0.051 |
|  | *nifD* | Nitrogenase molybdenum-iron protein alpha chain | 3.175 | 8.226 | 0.3 | 0.276 |
|  | *nifF* | Flavodoxin B | 2.281 | 1.818 | -0.1 | 0.503 |
|  | *nifH* | Nitrogenase iron protein | 2.706 | 2.281 | -0.2 | 0.559 |
| ——————————————————————————————————————————————————————— | | | | | | |

**Table S2.** Continued.

| ——————————————————————————————————————————————————————— | | | | | | | |
| --- | --- | --- | --- | --- | --- | --- | --- |
|  | | *nifH1* | Nitrogenase iron protein 1 | 2.369 | 4.790 | 0.1 | 0.301 |
|  | | *nifK* | Nitrogenase molybdenum-iron protein beta chain | 3.005 | 8.940 | 0.3 | 0.214 |
|  | | *nifL* | Nitrogen fixation regulatory protein | 0.584 | 0.920 | 0.5 | 0.097 |
|  | | *nifW* | Nitrogenase-stabilizing/protective protein NifW | 0.097 | 0.186 | 0.8 | 0.088 |
| Anammox | | | | | |  |  |
|  | |  | Hydrazine synthase subunit beta | 0.768 | 3.051 | 0.9 | 0.003 |
|  | |  | Hydrazine synthase subunit gamma | 0.058 | 0.261 | 0.2 | 0.468 |
| Denitrification | | | | | |  |  |
|  | Nitrite reductase | | |  |  |  |  |
|  | | *nirK* | Copper-containing Nitrite reductase | 5.409 | 2.593 | -1.2 | **0.005** |
|  | | *nirB* | **Cytochrome c-551** | 0.849 | 0.793 | 0.1 | 0.650 |
|  | | *nirM* | **Cytochrome c-551** | 1.055 | 0.821 | 0.1 | 0.795 |
|  | | *nirQ* | **Denitrification regulatory protein NirQ** | 8.640 | 10.311 | 0.1 | 0.834 |
|  | | *nirS* | Nitrite reductase | 11.296 | 8.136 | -0.2 | 0.448 |
|  | | *nirT* | Denitrification.system.component NirT | 0.667 | 0.545 | 0.3 | 0.301 |
|  | | *aniA* | Copper-containing nitrite reductases (*Neisseria*) | 8.148 | 6.929 | 0.1 | 0.824 |
|  | Nitric oxide reductase | | |  |  |  |  |
|  | | *norB* | Nitric oxide reductase subunit B | Xx | xx |  |  |
|  | | *norC* | Nitric oxide reductase subunit C | 2.101 | 1.554 | -0.1 | 0.731 |
|  | | *norG* | **HTH-type transcriptional regulator NorG** | 13.112 | 9.663 | -0.6 | 0.096 |
|  | | *norR* | Anaerobic Nitric oxide reductase transcription regulator NorR | 102.60 | 233.80 | 0.9 | **0.008** |
|  | | *norR1* | Nitric oxide reductase transcription regulator NorR1 | 0.035 | 0.104 | 0.2 | 0.539 |
|  | | *norR2* | Nitric oxide reductase transcription regulator NorR2 | 7.716 | 4.109 | -0.8 | **0.019** |
|  | | *norV* | Anaerobic Nitric oxide reductase flavorubredoxin | 12.370 | 17.810 | 0.0 | 0.933 |
|  | | *norW* | Nitric oxide reductase FlRd-NAD(+) reductase | 12.197 | 26.225 | 0.7 | **0.019** |
|  | Nitrous oxide reductase | | |  |  |  |  |
|  | | *nosD* | putative ABC transporter binding protein NosD | 7.208 | 8.520 | 0.2 | 0.600 |
|  | | *nosF* | putative ABC transporter ATP-binding protein NosF | 3.597 | 6.141 | 0.4 | 0.106 |
|  | | *nosL* | Copper-binding lipoprotein NosL | 2.148 | 1.934 | -0.1 | 0.854 |
|  | | *nosY* | putative ABC transporter permease protein NosY | 3.149 | 2.879 | -0.2 | 0.496 |
|  | | *nosZ* | Nitrous-oxide reductase | 12.246 | 15.399 | 0.2 | 0.495 |
|  | Nitrous oxide synthase | | |  |  |  |  |
|  | | *nos* | Nitric oxide synthase oxygenase | 0.110 | 0.384 | 0.2 | 0.420 |
| ——————————————————————————————————————————————————————— | | | | | | | |

**Table S2.** Continued

| ——————————————————————————————————————————————————————— |
| --- |

^a^ The effect size, which is defined as the difference between groups divided by the maximum dispersion within group A or B, was calculated with the ALDEx2 package (v: 1.21.1) using the aldex.ttest argument (Gloor et al., 2020). A positive value indicates that the relative abundance of the gene was higher after 74 days of composting compared its relative abundance in the cow manure at the onset of the experiment, while a negative value indicates the opposite, ^b^ The expected *p* value of the Kruskal-Wallis test as calculated with the ALDEx2 package (v: 1.21.1) using the aldex.ttest argument (Gloor et al., 2020), ND: Not detected, d p values in bold are significant at p < 0.05.

| ——————————————————————————————————————————————————————— |
| --- |
